# Age-related changes in adiposity and cardiometabolic disease risk: a longitudinal and prospective study in the UK Biobank

**DOI:** 10.1038/s41366-026-02168-2

**Published:** 2026-07-15

**Authors:** Mathias Rask-Andersen, Valeria Lo Faro, Torgny Karlsson, Åsa Johansson

**Affiliations:** 1 Department of Immunology, Genetics and Pathology, Science for Life Laboratory Uppsala University, Uppsala, Sweden; 2 Division of Clinical Epidemiology, Department of Medicine Solna, Karolinska Institutet, Stockholm, Sweden; 3 Uppsala University Future Institutes (UUniFI), Uppsala University, Uppsala, Sweden

**Keywords:** Risk factors, Medical research

## Abstract

**Background/Objectives:**

Body mass index (BMI) is widely used to assess obesity-related disease risk but does not capture important aspects of fat distribution and ectopic fat deposition. Here we assess age-related differences in magnetic resonance imaging (MRI)-derived body composition measures and their relevance for cardiometabolic disease risk.

**Subjects/Methods:**

We analysed 36,831 UK Biobank participants (45–82 years), with MRI-derived measures, including 1,688 participants with longitudinal measures. Nine MRI-derived traits and five cardiometabolic outcomes were analysed: type 2 diabetes (T2DM), hypertension, hyperlipidaemia, myocardial infarction, and cerebral infarction. Cox regression was used to estimate the association between adiposity traits and clinical outcomes and stepwise Cox regression to determine the relative importance of the variables. Significant associations were validated using univariable and multivariable Mendelian randomization (MR).

**Results:**

At the initial imaging session, several MRI-derived traits, including visceral adipose tissue (VAT) volume and pancreatic and thigh muscle fat infiltration, were higher with age at assessment and increased within participants between imaging timepoints in the longitudinal sub-cohort, despite similar BMI. In contrast, liver and pancreas volume were lower in older participants. All MRI-derived traits were associated with at least one clinical outcome. However, after adjustment for multiple testing and for BMI, only T2DM and hyperlipidemia remained significant. Including MRI-derived traits alongside BMI improved risk prediction, particularly in males. VAT showed the strongest association and accounted for a substantial proportion of the T2DM risk traditionally attributed to BMI in both females and males, as supported by multivariable MR.

**Conclusions:**

Ageing is associated with progressive, depot-specific redistribution of body fat with increasing accumulation in metabolically harmful depots later in life. MRI-derived adiposity measures provide important insight into these risk-relevant patterns. Future clinical translation may depend on more scalable tools or biological markers that capture visceral and ectopic fat accumulation and improve cardiometabolic risk assessment beyond BMI.

## Introduction

Obesity and overweight are well-known risk factors for disease and mortality [1, 2], and the ongoing global rise in obesity rates remains a significant concern [3]. The distribution of adipose tissue across various body depots and ectopic deposition within organs and muscles plays a critical role in obesity-associated disease risk. Accurate and biologically informative measures of adiposity are therefore essential for understanding obesity-related disease mechanisms and can also provide insights into heterogeneity in obesity-associated risk [4].

Where fat is stored may be more relevant for disease risk than total adiposity alone. Visceral adipose tissue (VAT) is a well-known risk factor for cardiovascular disease and cancer, which correlates strongly with total body fat [4, 5]. Myosteatosis, or fat infiltration in the thigh muscles, has also been linked to increased disease risk and mortality [6], while subcutaneous fat and fat accumulation in the waist and lower extremities is associated with lower disease risk [7]. Accurate and biologically informative measures of adiposity are therefore essential for understanding obesity-related disease mechanisms and can also provide insights into heterogeneity in obesity-associated risk [8].

Although magnetic resonance imaging (MRI) and computer tomography (CT) are able to provide detailed assessment of body composition in vivo, their high cost and complexity limit large-scale use. BMI is therefore often used as a proxy for adiposity, despite failing to account for fat distribution and ectopic fat. Imaging data also require further processing to produce quantitative estimates, which complicates large-scale epidemiological studies. Consequently, prior imaging studies are often limited by small sample sizes and a lack of prospective data, and large-scale longitudinal MRI-based studies remain scarce.

Characterising how adiposity changes across the life course has predominantly relied on easily accessible anthropometric traits. BMI increases until middle age and then declines in old age [9], while waist circumference (WC) and waist-to-hip ratio (WHR) continue to increase with age [10]. MRI and CT studies have further suggested that VAT, liver fat, and intramuscular fat increase with age, whereas subcutaneous fat in the lower body often declines or redistributes, patterns that are not fully captured by BMI or waist measures [11, 12]. However, most imaging studies have been relatively small, cross-sectional, and rarely capture longitudinal changes across multiple depots simultaneously. This highlights the need for large-scale longitudinal MRI-based studies to more precisely characterise depot-specific adiposity trajectories and their links to cardiometabolic disease.

**Fig. 1:**
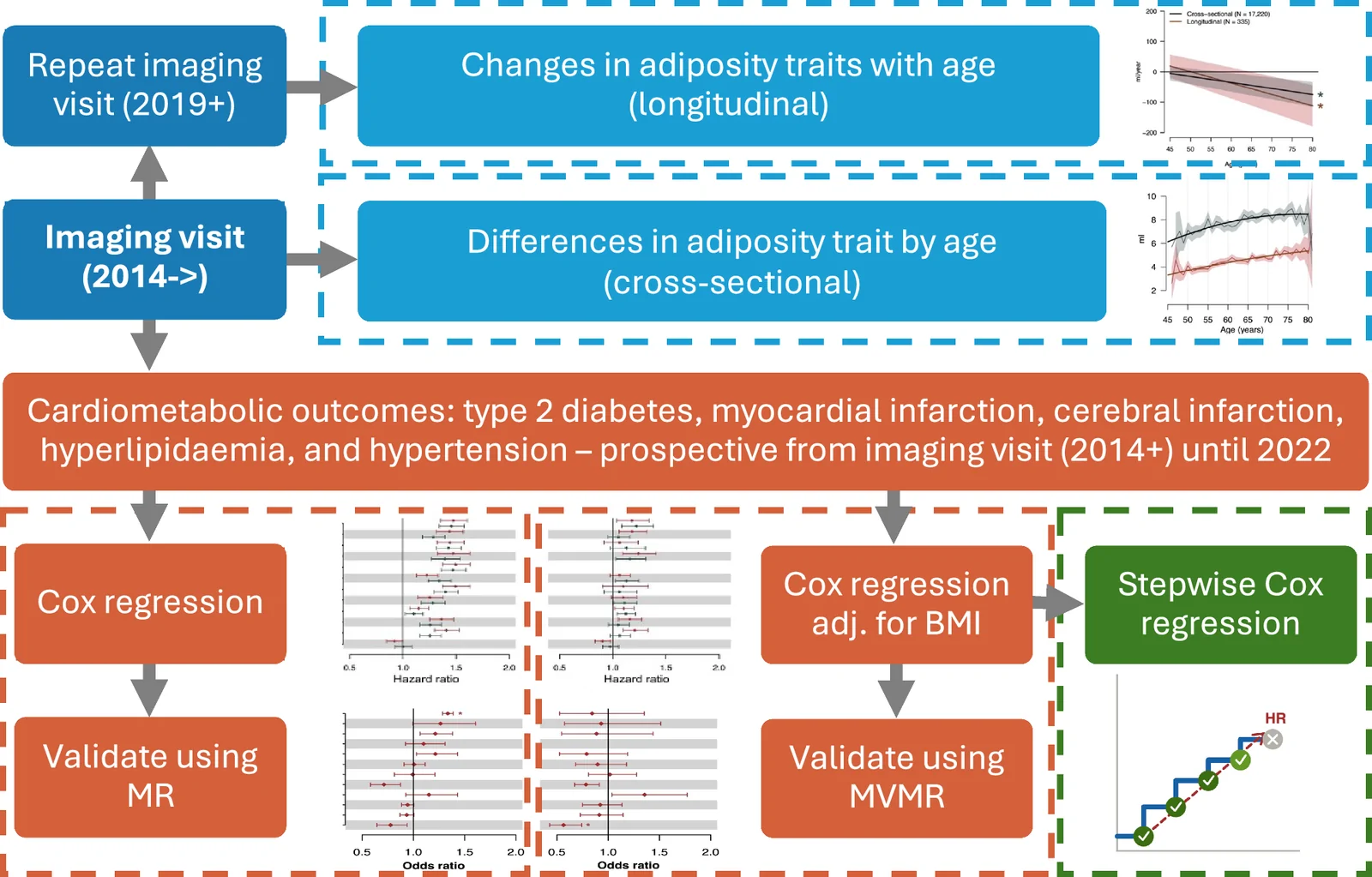
Overview of the study design linking adiposity traits to cardiometabolic outcomes. Imaging-derived adiposity traits were assessed at the baseline imaging visit (2014 → ) and, when available, at the repeat visit (2019 + ). Cross-sectional analyses examined age-related differences in adiposity traits at baseline, while longitudinal analyses quantified within-individual changes over time. Cardiometabolic outcomes, including type 2 diabetes, myocardial infarction, cerebral infarction, hyperlipidaemia, and hypertension, were followed prospectively from the baseline imaging visit until 2022. Associations between adiposity traits and incident cardiometabolic outcomes were evaluated using Cox regression models. Analyses were performed both unadjusted and adjusted for BMI, with downstream validation using univariable Mendelian randomization (MR) and multivariable MR (MVMR). Stepwise Cox regression to evaluate the relative contribution of different adiposity traits to cardiometabolic risk.

In this study, we aimed to characterise age-related differences in MRI-derived adiposity traits and to assess their relevance for cardiometabolic disease risk. While these diseases have heterogeneous manifestations, they partly share common pathophysiological pathways linked to adipose tissue dysfunction, including insulin resistance, chronic inflammation, and ectopic fat accumulation. We hypothesised that MRI-based measures of fat distribution and ectopic fat would capture age-related variation in adiposity more precisely than conventional anthropometric measures and thereby provide deeper insight into disease risk. To test this, we analysed MRI-derived adiposity data from 36,831 UK Biobank participants, including longitudinal data for up to 1,688 participants and examined VAT, subcutaneous abdominal fat (SAT), liver and pancreas fat, and thigh muscle fat infiltration and their contributions to the risk of type 2 diabetes (T2DM), hypertension, hyperlipidaemia, myocardial infarction, and cerebral infarction (Fig. 1).

## Methods

### UK Biobank cohort

The UK Biobank (UKB) is a population-based cohort study involving approximately 500,000 UK residents aged 37–73 years at recruitment between 2006 and 2010. Ethnic background was assessed using a touchscreen questionnaire at the first assessment centre visit (Data Field 21000). In this study, approximately 91% of participants who self-identified as White British in the UK Biobank were included to minimise confounding due to population stratification. For more info see Supplementary Information.

### Ethics approval

All participants in the UKB provided written informed consent, and the study was conducted in compliance with the principles outlined in the Declaration of Helsinki. Ethical approval for the UK Biobank project was granted by the National Research Ethics Committee (11/NW/0382), allowing bona fide researchers to conduct research on UK Biobank data without separate ethical approval. Approval for the use of data was granted for this project by the UK Biobank access subcommittee (application number: 15152). Ethical approval was also granted for storage and analysis of UK Biobank data in Sweden by the Swedish Ethical Review Authority (dnr: 2020-04415 and 2024-07639-02).

### MRI-based phenotypes

MRI imaging in UK Biobank was initiated in 2014 and is currently ongoing, aiming to reach 100,000 participants with follow-up imaging in 70,000 (see Supplementary Information for more information). When our study was initiated, raw imaging data was available for ~40,000 participants at initial imaging and ~6,969 at follow-up (data accessed 22 May 2023). However, for this study we used the quantitative body composition measurements that had been derived from raw imaging data and supplied to UK Biobank by two sources: AMRA medical AB (Linköping, Sweden) [13] and Calico Life Sciences LLC (South San Francisco, USA) [6, 14]. The total sample size in this study is therefore 36,831 white British UK Biobank participants (aged 45–82 at the first imaging), with MRI derived phenotypes from the initial imaging visit, of whom 1,688 have longitudinal MRI-derived phenotypes. Data used in this study included abdominal body composition (subcutaneous and VAT volumes: Calico, data fields 21085-6), liver (Calico, data field 21088-9) and pancreas (Calico, data fields 21087 and 21090) volume and fat content, and anterior and posterior thigh muscle composition (AMRA, data fields 22403-6).

### Age-related differences/changes in adiposity traits

For the cross-sectional cohort, age-specific averages were calculated for each year of age at the initial imaging visit, separately for females and males. Second-degree polynomial models were fitted to the data using linear regression with first and second order terms in age to describe age-related differences in anthropometric and imaging-derived traits (see Supplementary Information). To estimate the age at which each trait reached a plateau, we derived the turning point of the fitted polynomial functions. Uncertainty in the inferred plateau ages was quantified using parametric Bootstrap simulations as described in the Supplementary Information. For the longitudinal data, we assessed changes over time by calculating average rates of change (Supplementary Fig. S1). Linear models with a first order term in age were fitted to the data to describe age-related rates of change (Supplementary Information).

### Clinical outcomes

Date of first diagnosis of hyperlipidaemia, T2DM, hypertension, myocardial infarction, or cerebral infarction, had been derived from primary care, hospital, death records, and self-reports, mapped to three-character ICD10 codes. The outcomes used in our study were made available by UK Biobank as data-fields: 130708 (E11: non-insulin-dependent diabetes mellitus), 131286 (I10: essential hypertension),131298 (I21: acute myocardial infarction) and 131366 (I63, cerebral infarction). For hyperlipidaemia we used only the hospital inpatient data (data-field 41270; ICD10 code E78: disorders of lipoprotein metabolism and other lipidaemias). Participants with a prior diagnosis of type T2DM, myocardial infarction, cerebral infarction, hypertension, or cancer were excluded from longitudinal analyses. Incidence rates with corresponding 95% CIs were estimated using the IRCI function in the fmsb R package. When comparing incidence rates between females and males, p-values were calculated from two-sided Z-tests (Wald method) assuming asymptotic normality. The Z-statistic was calculated as the difference in incidence rates divided by the square root of the sum of squared standard errors, where sex-specific standard errors were estimated assuming the incidence rates follow scaled Poisson distributions. These assumptions are appropriate given the large sample size, and the incidence rates approximate the conditions required for the Z-tests.

### Cox-proportional hazards modelling

Trait associated disease risk was estimated using Cox proportional hazards modelling in R version 3.6.0 [15] using the ‘survival’ R package version 3.5-5 [16]. Participants with the disease under investigation at the start of follow-up were excluded, as well as participants with missing information on any of the covariates. Patients were followed from their first MRI session (2014 onwards) until diagnosis of the disease, death, or the end of follow-up (November 2022). Death was treated as a censoring event; no other disease outcomes were treated as competing risks and no additional competing risk framework was applied. Cox proportional hazards models were fitted using the ‘coxph’ function in R, with survival as the response variable, including age at initial assessment, disease status (1 = diagnosis, 0 = no diagnosis), and age at follow-up in females and males separately. Age was used as the primary time-scale to prevent age-related confounding [17]. Adiposity traits at baseline were the exposures, with year of birth, and smoking as covariates. We aimed to keep the model parsimonious, using a limited set of well-validated covariates as described in the Supplementary Information. To ensure comparability of hazard ratios (HRs) between sexes and between different MRI traits and measuring methods, all MRI traits were rank-based inverse normal transformed (INT) separately by sex. Additional models included BMI to estimate BMI-independent effects of other adiposity measures. Bonferroni adjustment was performed to account for the family-wise error rate and *P*-values < 4.17*10^-4^ (for 120 tests: 12 adiposity traits tested for association with five outcomes in both female and male participants) were considered significant. Proportional hazards assumptions for all adiposity traits were assessed using scaled Schoenfeld residuals generated and visualised using the ‘cox.zph’ and ‘ggcoxzph’ functions in R.

### Stepwise Cox proportional hazards regression

Stepwise Cox proportional hazards regression was performed using the ‘stepwiseCox’ function in the ‘StepReg’ R-package v1.4.4, with bidirectional selection to identify key variables for disease risk. AIC determined variable inclusion/exclusion [18]. The analysis was done separately for females and males using a 50% random subset (training set). Selected variables were tested for discriminatory power in the other 50% (testing set) using Gönen and Heller’s concordance indices (GHCIs) [19], with the ‘survAUC’ package in R [20]. Four models were compared: 1) base model (birth year, smoking); 2) BMI added; 3) stepwise model with selected variables; and 4) full model (selected variables with BMI added).

### Univariable Mendelian Randomization analyses

Mendelian randomization (MR) leverages genetic variants as instrumental variables to assess causal relationships which minimises confounding and reverse causation biases that often are present in observational studies. Two-sample MR was performed to assess the causal effects of adiposity traits on disease outcomes. Genetic instruments for 12 adiposity traits were identified using Plink v2 [21] from UK Biobank GWAS and publicly available datasets (Table S1) as described in the Supplementary Information.

The inverse-variance weighted (IVW) method was used as the primary method for causal association analysis. MR relies on three core assumptions: relevance, independence, and exclusion restriction. The last assumption, also referred to as no horizontal pleiotropy assumption, was assessed via several sensitivity analyses to ensure the robustness of the results, including both random- and fixed-effect IVW and MR-Egger. The first assumption was satisfied by accepting only genome-wide significant variants as genetic instruments. Univariable MR analyses were conducted using the ‘TwoSampleMR’ package in R [22].

### Proportion of association explained by adiposity traits

We assessed the extent to which the association between BMI and disease risk was explained by other adiposity phenotypes using the difference-in-coefficients method [23]. Cox proportional hazards models were built for BMI alone and for BMI with each adiposity trait. HR estimates for BMI were compared between the base and extended models. Sobel test was used to assess whether the association between BMI and the outcome was attenuated after inclusion of the adiposity trait in the model and two-sided *P*-values reported. Only outcomes where adiposity traits significantly affected disease rates after adjusting for BMI were evaluated in these analyses.

### Multivariable Mendelian Randomization

As an independent extension of the previous analyses, multivariable MR (MVMR) was performed with the ‘MendelianRandomization’ R-package [24] to explore how other adiposity traits contribute to the associations between BMI-associated genetic instruments and disease risk [25]. Details of the MVMR implementation, including instrument selection, conditional F-statistics, and the estimation of indirect effects using a difference-in-coefficients approach, along with the use of random-effects inverse-variance weighted models and Sobel tests, are provided in the Supplementary Information.

## Results

### Females and males exhibit distinct adiposity profiles

The mean age at initial MRI was 63.9 ± 7.3 years and MRI-derived variables were available for 36,831 participants (Table 1). For participants attending the second imaging session, the average time between sessions was 2.7 ± 1.2 years. Participants who underwent MRI tended to be younger and healthier than the full UK Biobank cohort at baseline (Table S2), likely due to selective participation. Females had lower average weight, height, BMI, waist circumference (WC), WHR, VAT, liver and pancreas fat percentages, and thigh fat-free muscle volume (FFMV) compared to males (Table 1). However, females had higher SAT volumes and greater thigh muscle fat infiltration (MFI).

**Table 1:** Characteristics of the UK Biobank participants with MRI data from the initial and 2nd imaging sessions.

	Females							Males						
	Initial imaging (*N*)	Mean ± SD	P (F-test)	2nd imaging (*N*)^#^	Mean ± SD	Change (Mean ± SD)	*P* (t-test)	Initial imaging (*N*)	(Mean ± SD)	P (F-test)	2nd imaging (N)	Mean ± SD	Change (Mean ± SD)	*P* (t-test)
Age (years)	19,034	63.3 ± 7.4		2427	63.9 ± 7.1	2.7 ± 1.2	***	17,797	64.7 ± 7.6		2291	65.6 ± 7.5	2.7 ± 1.2	***
Year of birth (year)	19,034	1954 ± 7		2427	1955 ± 7.2			17,797	1952 ± 8		2291	1954 ± 7.6		
Height (cm)	18,436	162.8 ± 6.2	***	2411	163.0 ± 6.3	-0.2 ± 1.2		17,312	176.2 ± 6.6	***	2276	176.4 ± 6.7	−0.2 ± 1.2	
Weight (kg)	18,425	69.1 ± 13.0	***	2408	68.9 ± 13.1	-0.021 ± 4.0		17,289	83.9 ± 13.4	***	2273	83.5 ± 13.2	−0.356 ± 4.0	
BMI (kg/m2)	18,399	26.1 ± 4.7	*	2408	25.9 ± 4.8	0.05 ± 1.54		17,279	27.0 ± 3.9	***	2273	26.8 ± 3.9	−0.06 ± 1.30	
Waist (cm)	18,460	82.8 ± 11.8	***	2410	83.5 ± 11.6	1.0 ± 6.8	**	17,321	94.5 ± 10.7	***	2276	94.7 ± 10.6	0.6 ± 6.0	
Hip (cm)	18,460	101.0 ± 9.8	***	2410	99.9 ± 9.6	-0.8 ± 5.8	**	17,323	101.0 ± 7.3	***	2276	99.6 ± 7.1	-0.9 ± 5.3	***
Waist-to-hip ratio	18,459	0.82 ± 0.07	***	2410	0.84 ± 0.07	0.02 ± 0.06	***	17,321	0.93 ± 0.06	***	2276	0.95 ± 0.07	0.02 ± 0.05	***
VAT (l)	18,400	2.84 ± 1.57	***	902	2.85 ± 1.63	0.19 ± 0.57	*	17,191	5.20 ± 2.36	***	780	5.28 ± 2.38	0.129 ± 0.951	
SAT (l)	18,400	9.9 ± 4.4	***	902	9.9 ± 4.7	0.224 ± 1.688		17,191	7.0 ± 3.3	***	780	7.2 ± 3.2	0.081 ± 1.264	
VAT-SAT ratio	18,400	0.28 ± 0.10	***	902	0.28 ± 0.10	0.01 ± 0.03	**	17,191	0.77 ± 0.25	***	780	0.76 ± 0.24	0.01 ± 0.09	
Pancreas volume (mL)	17,763	55 ± 16	***	242	59 ± 14	0.3 ± 12.5		16,630	63 ± 18	***	209	66 ± 16	−0.7 ± 12.0	
Pancreas fat fraction (%)	13,855	8.6 ± 6.9	***	233	7.8 ± 6.5	1.2 ± 5.1	*	13,087	13.0 ± 8.8	***	202	13.5 ± 9.2	0.7 ± 6.3	
Liver volume (mL)	18,400	1311 ± 269	***	902	1322 ± 275	−13 ± 127		17,191	1538 ± 318	***	780	1534 ± 320	−35 ± 178	*
Liver fat fraction (%)	14,292	4.2 ± 4.6	***	902	4.3 ± 4.5	0.4 ± 2.7		13,440	5.6 ± 5.1	***	786	6.0 ± 5.4	0.2 ± 3.5	
Anterior thigh FFMV (l)	18,822	2.68 ± 0.44	***	407	2.69 ± 0.46	−0.08 ± 0.13	*	17,174	4.20 ± 0.69	***	336	4.25 ± 0.68	−0.12 ± 0.22	*
Anterior thigh MFI (%)	18,788	11.8 ± 2.8	***	407	11.6 ± 2.9	0.5 ± 0.7	*	17,136	10.3 ± 2.6	***	336	9.9 ± 2.6	0.4 ± 0.7	*
Posterior thigh FFMV (l)	18,822	5.51 ± 0.75	***	407	5.62 ± 0.80	−0.06 ± 0.21		17,220	8.11 ± 1.11	***	336	8.24 ± 1.11	−0.11 ± 0.29	
Posterior thigh MFI (%)	18,789	17.3 ± 3.5	***	407	17.2 ± 3.7	0.5 ± 0.9	*	17,182	15.6 ± 3.5	***	336	15.5 ± 3.4	0.5 ± 0.8	

Characteristics are presented for all participants who underwent initial and follow-up magnetic resonance imaging. The F-test was used to test for cross-sectionally age-related differences in the traits, assuming a second-degree polynomial as a model for the effect of age on the trait (see ‘Methods’). Student’s t-test were used to test for longitudinal changes between timepoints among participants with data from two imaging sessions. ^*^ = *P* < 0.05, ^**^ = *P* < 0.01, ^***^ = *P* < 0.001. # - Only a subset of participants who attended the repeat imaging visit had derived MRI phenotypes available.

*VAT* visceral adipose tissue, *SAT* subcutaneous adipose tissue, *MFI* muscle fat infiltration, *FFMV* fat-free muscle volume.

### Distinct age-related patterns in adiposity and harmful fat depots

**Fig. 2:**
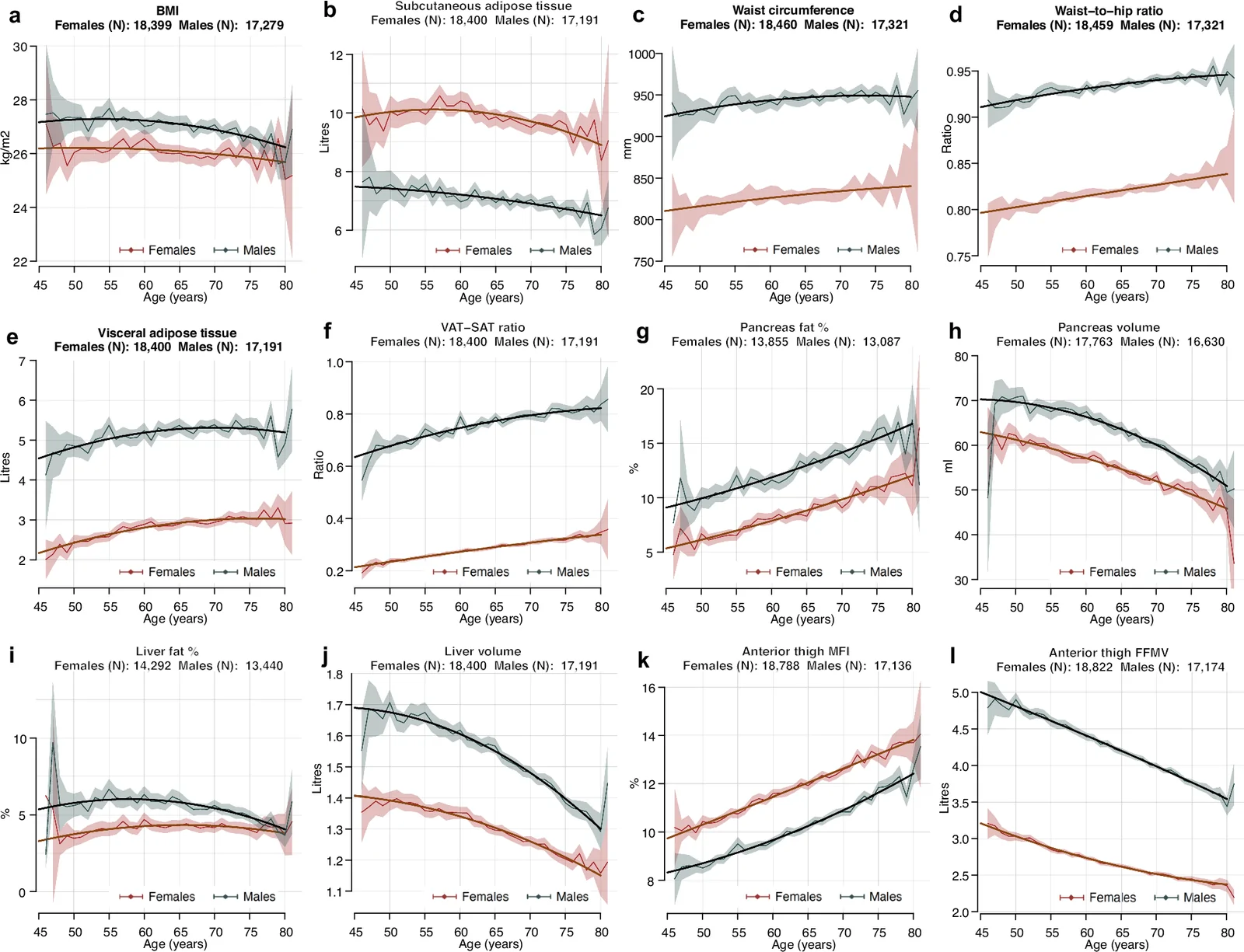
Age related trends in anthropometric and MRI derived measurements in the cross-sectional cohort. Data are presented separately for females (red) and males (green). Cross-sectional mean values at initial imaging visit are shown for each year of age with the thin solid lines connecting age-specific means and the shaded areas representing 95% confidence intervals. Second-degree polynomials were fitted to the data using linear regression modelling in R (thick solid lines). The panels show: (**a**) body mass index (BMI); (**b**) subcutaneous adipose tissue (SAT); (**c**) waist circumference; (**d**) waist-to-hip ratio; (**e**) visceral adipose tissue (VAT); (**f**) VAT–SAT ratio; (**g**) pancreas fat %; (**h**) pancreas volume; (**i**) liver fat %; (**j**) liver volume; (**k**) anterior thigh muscle fat infiltration (MFI); and (**l**) anterior thigh fat-free muscle volume (FFMV).

Cross-sectional data showed strong, generally non-linear, age-related patterns in adiposity traits (Fig. 2 and Supplementary Data 1), which were largely confirmed in the longitudinal cohort (Figs. S2 and S3). While BMI and SAT (Fig. 2a, b) were lower in older age groups, WC, WHR, VAT, and the VAT/SAT ratio (Fig. 2c–f) were higher when comparing across age groups in these cross-sectional analyses. Pancreas fat was higher (Fig. 2g) and pancreas volume lower (Fig. 2h) in older participants. Liver fat was similar in the youngest and oldest groups (Fig. 2i), whereas liver volume was substantially lower in older participants (Fig. 2j). MFI was higher (Fig. 2k) and FFMV lower (Fig. 2l) in older participants. These patterns were broadly consistent with observations from the longitudinal data. In the cross-sectional cohort, VAT plateaued at age 76.0 (95% CI 71.9–84.9) in females and 70.1 (68.0–75.8) in males and liver fat percentage plateaued at age 65.4 (64.8–67.5) in females and 57.9 (54.2–59.5) in males (Supplementary Data 1). Longitudinal data supported the presence of these plateaus, but the observations were too few to infer age estimates (Figs. S4–S8).

Pancreatic fat percentage was positively associated with age in the cross-sectional cohort alongside a negative association with pancreatic volume. We considered whether age-related decreases in organ volume could result in higher measured fat percentages without reflecting true ectopic fat accumulation. However, when calculating pancreatic fat volume, by multiplying fat percentage by total pancreatic volume, we observed that total pancreatic fat amount was consistently higher with older age at assessment (Fig. S8).

Due to small sample sizes in other ethnic groups, statistical analyses of age-related adiposity differences were not feasible. However, trends in these groups were similar to those in the participants with white British ancestry (Table S3 and Figs. S9–S12).

### Adiposity drives disease risk across middle and old age

**Fig. 3:**
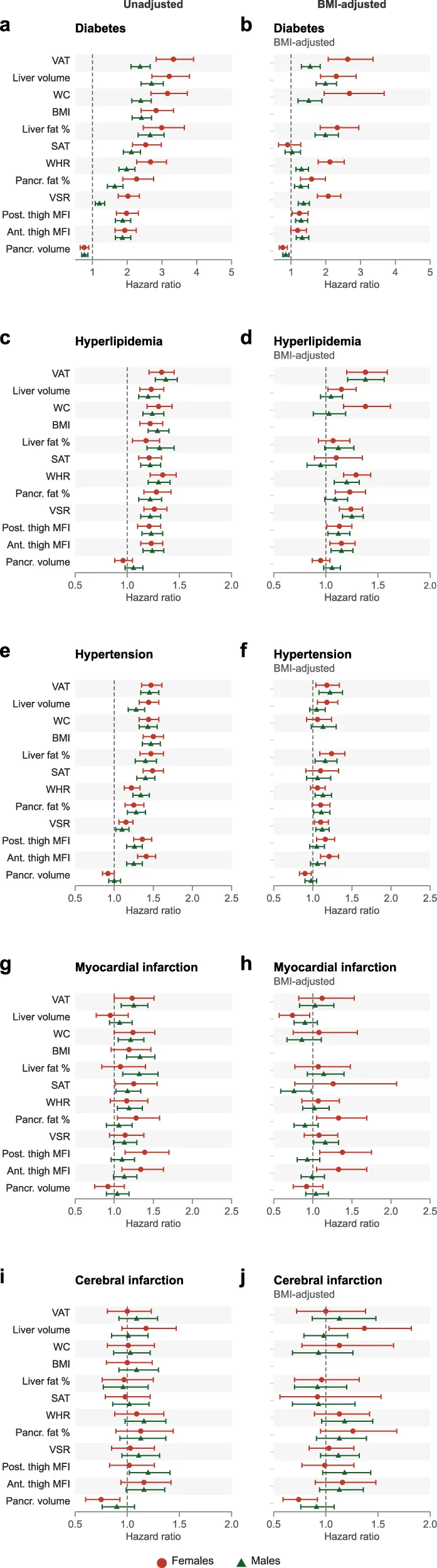
Hazard ratios for cardiometabolic outcomes associated with different adiposity measures, with and without adjustment for BMI. Cox proportional hazards modelling was used to estimate the hazard ratio (HR) and 95% confidence intervals (CI) of adiposity-related traits on cardiometabolic outcomes in females (red), males (green). All traits were normalised by rank-based inverse normal transformation in females and males separately and results for traits on their original scales are provided in Supplementary Data 2. HRs that are presented here therefore represent the increase in event rate that is associated with a one standard deviation increase in the trait in either males or females. Smoking, year of birth and 15 genetic principal components were included as covariates. The panels show: (**a–b**) type 2 diabetes mellitus (T2DM), without (**a**) and with (**b**) adjustment for BMI; (**c–d**) hyperlipidemia, without (**c**) and with (**d**) adjustment for BMI; (**e–f**) hypertension, without (**e**) and with (**f**) adjustment for BMI; (**g–h**) myocardial infarction, without (**g**) and with (**h**) adjustment for BMI; and (**i–j**) cerebral infarction, without (**i**) and with (**j**) adjustment for BMI. Abbreviations: VAT visceral adipose tissue, SAT subcutaneous adipose tissue, BMI body mass index, MFI muscle fat infiltration, Post. posterior, Ant. anterior.

Among the 17,797 males and 19,034 females, 341 (0.92%) were diagnosed with myocardial infarction, 244 (0.66%) with ischaemic stroke, 503 (1.37%) with T2DM, 1345 (3.65%) with hypertension, and 1189 (3.23%) with hyperlipidaemia between the first imaging visit and the end of follow-up, with higher incidence rates in males (Table 2). The average follow-up ranged from 4.4 to 5.1 years depending on the outcome (Supplementary Data 2). All MRI-derived traits were at least nominally associated with increased risks for T2DM and hyperlipidaemia (Fig. 2). A vast majority were also significant when Bonferroni adjustment for multiple testing was applied (Supplementary Data 2) and when adjusting for BMI (Supplementary Data 3), which suggests that they contribute towards disease risk on top of BMI. The strongest association was between VAT and T2DM (Fig. 3a) in females (HR = 3.33, 95% CI 2.83–3.91), which was only slightly attenuated (Fig. 3b) after adjusting for BMI (HR = 2.63, 95% CI 2.07–3.36). Similar, but weaker, effects were seen in males (Fig. 3a, b). VAT showed a strong association with hyperlipidaemia (Fig. 3c), with slightly larger HRs after adjusting for BMI (Fig. 3d).

**Table 2:** Prevalent and incident cases and incidence rates of cardiometabolic disease after the first MRI assessment.

	Females				Males				Difference in IR between females & males
	Total (N)	Prevalent cases (N)	Incident cases (N)	IR (95% CI)^a^	Total (N)	Prevalent cases (N)	Incident cases (N)	IR (95% CI)	*P*-value
T2DM	19,034	410	175	184.3 (157.0 - 211.6)	17,797	837	328	383.7 (342.2 - 425.2)	3.7 × 10^−15^
Hypertension	19,034	4260	621	848.3 (781.6 - 915.1)	17,797	6182	724	1281.6 (1188.3 - 1375.0)	1.4 × 10^−13^
Myocardial infarction	19,034	144	101	104.4 (84.0 - 124.8)	17,797	683	241	278.0 (242.9 - 313.1)	5.0 × 10^−17^
Hyperlipidemia	19,034	2738	520	639.7 (584.7 - 694.6)	17,797	4733	669	1047.4 (968.1 - 1126.8)	1.3 × 10^−16^
Cerebral infarction	19,034	40	95	97.6 (78.0 - 117.3)	17,797	128	149	166.0 (139.3 - 192.6)	5.2 × 10^−5^

For each outcome, female and male participants are shown separately. Total (*N*) = number of participants with MRI data, Prevalent cases (*N*) = number of participants diagnosed with the condition at or before baseline MRI, Incident cases (*N*) = new diagnoses after baseline MRI, and IR = incidence rate per 100,000 person-years with 95% confidence interval (CI). *P*-values are from Z-tests comparing incidence rates between females and males. *P* < 0.05 was considered statistically significant.

Several of the MRI-traits were also strongly associated with hypertension (Fig. 3e), however these associations were markedly attenuated after adjusting for BMI (Fig. 3f), indicating that they largely capture the same effect as BMI. After multiple testing adjustment, no MRI-traits reached significance for myocardial infarction or cerebral infarction (Supplementary Data 2). However, some traits were nominally significant (Fig. 3g–j). For example, pancreas volume was associated with a reduced risk of stroke (Fig. 3i) in females (HR = 0.75, 95% CI 0.60–0.93), and this association remained after adjustment for BMI (Fig. 3j) with similar results observed for T2DM (Fig. 3a, b). Although the association did not withstand correction for multiple testing, it may still be important given the known link between pancreatic atrophy and increased disease risk [26, 27].

Schoenfeld residual analyses showed nominal deviations from the proportional hazards assumption in several models (nominal *P*-value < 0.05), including the association between VAT and T2DM risk in females (Fig. S13). However, none of these findings remained significant after correction for multiple testing.

### Univariable Mendelian randomization to determine causal effects of adiposity traits on disease

MR analyses provide an additional layer of evidence to support the significant Cox regression results outlined above for T2DM, hypertension and hyperlipidaemia. However, GWAS summary statistics were only available for males and females combined, which limited our ability to perform sex-stratified MR. While fixed-effect IVW estimates closely mirrored Cox regression results (Supplementary Data 4), random-effects MR models could not confirm all results (Supplementary Data 4). Significant heterogeneity was detected among the instruments in most analyses; to account for this, the random-effects IVW method were considered the primary results, as random effects models reduce the risk of underestimated standard errors and false positives. Therefore, only a limited number of findings from the Cox regression results were replicated after considering multiple testing adjustment (Fig. S14).

### Adiposity traits enhance predictive modelling for disease risk

Stepwise Cox proportional hazards modelling was used to identify which adiposity variables were most predictive of disease outcomes in females and males. Half of the cohort was used to train the models, and the other half for validation. Due to the absence of significant associations between the adiposity traits and cerebral infarction or myocardial infarction after multiple testing correction, as well as the lack of significant associations for hypertension after adjusting for BMI, these three outcomes were not included in these analyses.

Notably, BMI was not selected as a predictive variable for T2DM or hyperlipidaemia in either sex (Tables S4 and S5), which suggests BMI-related risk to be explained by specific fat depots or ectopic fat. Evaluation of the models’ discriminatory power showed that BMI improved predictive accuracy for T2DM and hyperlipidaemia in the validation subset. However, models that replace BMI with adiposity traits selected through stepwise modelling performed similarly or better. In males, T2DM prediction improved when BMI was replaced by liver fat percentage, liver volume, pancreas volume, and VSR, increasing the concordance index from 0.675 to 0.736 (Table 3).

**Table 3:** Discriminatory power of Cox proportional hazards models for T2DM and hyperlipidaemia.

Disease	Sex		Model	Model specification	GHCI	n
T2DM	Females		Base model	*Survival ~ smoking + year of birth*	0.811	6099
			Base + BMI	*Survival ~ smoking + year of birth* + *BMI*	0.844	6099
			Base + selected traits	*Survival ~ smoking + year of birth + visceral adipose tissue + pancreas volume + liver fat % + year of birth* + *WHR + liver volume*	0.849	6099
			Base + selected traits + BMI	*Survival ~ smoking + year of birth + visceral adipose tissue + pancreas volume + liver fat % + year of birth* + *WHR + liver volume* + *BMI*	0.851	6099
	Males		Base model	*Survival ~ smoking + year of birth*	0.567	5720
			Base + BMI	*Survival ~ smoking + year of birth* + *BMI*	0.675	5720
			Base + selected traits	*Survival ~ smoking + year of birth + liver fat % + liver volume + pancreas volume* + *VSR*	0.736	5720
			Base + selected traits + BMI	*Survival ~ smoking + year of birth + liver fat % + liver volume + pancreas volume* + *VSR* + *BMI*	0.738	5720
Hyperlipidemia	Females		Base model	*Survival ~ smoking + year of birth*	0.786	6099
			Base + BMI	*Survival ~ smoking + year of birth* + *BMI*	0.798	6099
			Base + selected traits	*Survival ~ smoking + year of birth + pancreas fat % + liver volume* + *VSR*	0.802	6099
			Base + selected traits + BMI	*Survival ~ smoking + year of birth + pancreas fat % + liver volume* + *VSR* + *BMI*	0.802	6099
	Males		Base model	*Survival ~ smoking + year of birth*	0.763	5720
			Base + BMI	*Survival ~ smoking + year of birth* + *BMI*	0.779	5720
			Base + selected traits	*Survival ~ smoking + year of birth* + *VAT* + *WHR* + *WC*	0.789	5720
			Base + selected traits + BMI	*Survival ~ smoking + year of birth* + *VAT* + *WHR* + *WC* + *BMI*	0.789	5720

Covariates were selected for inclusion in models through stepwise Cox proportional hazards modelling.

*GHCI* Gönen and Heller’s concordance index for Cox proportional hazards models, *BMI* body mass index, *WHR* waist-to-hip ratio, *WC* waist circumference, *VSR* VAT-SAT ratio.

### Difference-in-coefficients analysis reveal key roles for VAT and ectopic fat

Difference-in-coefficients analyses were performed to assess how BMI-associated effects on disease risk could be explained by other adiposity traits. Only T2DM and hyperlipidaemia were included, as no significant associations with the other outcomes were found after adjusting for BMI (Figs. 3 and S14). Several adiposity traits partly explain the association between BMI and T2DM (Sobel test *P*-value < 0.0033), including VAT, ectopic fat in the liver and pancreas, and liver volume in both sexes. WC and WHR were also significant in females (Table S6). VAT accounted for the largest proportion of the BMI effect on T2DM, with point estimates of 77% in females and 43% in males. For hyperlipidaemia, VAT appeared to account for up to 100% of the effect in females and 68% in males, while pancreatic fat percentage explained 71% of BMI’s effect in females (Table S7). However, it should be noted that these point estimates are associated with substantial uncertainty (Tables S6 and S7) and should therefore be interpreted with caution.

### Multivariable MR validation confirms the key role of VAT

To validate the difference-in-coefficients results, we used MVMR. Due to the lack of sex-stratified GWAS data, MVMR was conducted on sex-combined data. Conditional F-statistic indicated weak instrument bias found for all adiposity traits except VAT, liver volume, and anterior thigh MFI, so MVMR was restricted to these traits (Supplementary Data 5). In MVMR, the causal effect of VAT on T2DM was significant after adjusting for BMI, consisted with the Cox regression results. However, while the Sobel test confirmed that VAT contributes to part of BMI’s effect on T2DM (*P* = 0.012), the proportion of the effect explained by VAT was relatively small, estimated to only 11%. For the two other traits evaluated with MVMR, no significant proportion of BMI’s effect on T2DM was explained by anterior thigh MFI, nor was BMI’s effect on hyperlipidaemia significantly explained by liver volume (Supplementary Data 5), potentially due to limited statistical power.

## Discussion

Our findings show that abdominal adiposity and fat infiltration in muscles and pancreas increase with age, even as BMI plateaus in the early 50 s and subsequently declines. Importantly, our work extends previous research by systematically comparing multiple MRI-derived measures of adiposity. While earlier studies have reported that BMI stabilises or decreases with age, we provide direct evidence that visceral and ectopic fat depots continue to expand over time. This redistribution occurs independently of BMI and disproportionately affects metabolically harmful fat depots, including VAT and pancreatic fat. Taken together, these findings point to a progressively unfavourable adiposity profile with advancing age, which agrees with previous studies [28]. Our results also help explain why BMI becomes a less informative marker of cardiometabolic risk in older populations, provide mechanistic insight into the role of age-related fat redistribution in disease risk, and support the incorporation of alternative adiposity measures into risk prediction.

Age-related trends were consistent across both cross-sectional and longitudinal analyses and are in line with previous studies. In contrast to earlier studies that primarily relied on BMI, WC, BMI, WC, or WHR, our use of MRI-derived measures provides a more detailed and nuanced characterisation of adiposity-related risks. For example, while previous analyses in smaller subsets of the UK Biobank reported increases in VAT and muscle fat infiltration with age [29–34], our findings further reveal stable liver fat content across middle to old age, consistent with studies that have suggested stabilisation or decline after age 50 [35, 36]. For pancreatic fat and volume, the limited longitudinal sample size reduced statistical power; however, cross-sectional results were consistent with previous reports [37–39]. Notably, our longitudinal analyses showed higher rates of change for VAT, SAT, WC, and WHR, suggesting that cross-sectional studies may slightly underestimate the rate of abdominal fat accumulation with age.

Our observations also demonstrate the importance of ectopic fat deposition and organ volume in explaining a part of the BMI-associated risks. For example, stepwise Cox regression showed that BMI was not a significant predictor of T2DM or hyperlipidaemia when more precise adiposity traits were included. In agreement with this, difference-in-coefficients analyses that VAT explains a large proportion BMI-associated risks for T2DM and hyperlipidaemia. This highlights the limitations of BMI in estimating disease risk, as it does not capture fat distribution or ectopic fat deposition. MRI-derived adiposity measures, however, provide a more accurate assessment of pathogenic fat depots related to disease risk. This is consistent with previous studies suggesting that VAT and ectopic fat are more closely tied to cardiometabolic risk than BMI because they reflect metabolically adverse fat distribution and adipose tissue dysfunction rather than total fat mass [40]. When the storage capacity of subcutaneous adipose tissue is exceeded, excess lipids accumulate in visceral depots and non-adipose organs, contributing to lipotoxicity, insulin resistance, and increased hepatic glucose production [41]. VAT is also more metabolically active and is associated with chronic low-grade inflammation and altered secretion of signalling molecules from adipose tissue, which may contribute to atherosclerosis and cardiometabolic disease [42].

The close physiological link between visceral fat and the liver may help explain why VAT is more strongly related to metabolic risk than BMI, particularly through effects on hepatic insulin resistance and liver fat accumulation [43]. Similarly, pancreatic fat accumulation has been linked to impaired beta-cell function and increased T2DM risk, while liver fat is a well-established driver of dyslipidaemia and cardiometabolic disease independent of BMI [44]. In addition, reduced muscle quality, reflected by increased intramuscular fat, has been associated with insulin resistance and adverse metabolic outcomes [45]. Collectively, these mechanisms suggest that adipose tissue distribution and ectopic fat deposition, rather than total fat mass, are key determinants of cardiometabolic risk, consistent with MRI-derived adiposity traits being more informative than BMI in our study.

A major strength of this study is the combination of the large-scale imaging and genetic data available in UK Biobank, which allowed us to examine adiposity traits both observationally and through complementary MR analyses. A major limitation, however, is the limited number of participants with repeated MRI‑derived phenotypes, such that most age-related patterns are inferred from cross-sectional age differences rather than longitudinal within-person change. The available longitudinal data were broadly consistent with cross-sectional findings, but statistical analysis was limited by sample size. Another major limitation was the short follow-up time, which resulted in few incident cases for some outcomes (e.g. ischaemic stroke and myocardial infarction) and consequently limited statistical power to detect associations with MRI-derived phenotypes. Other limitations include the restriction to individuals of white British ancestry and a participation (healthy volunteer) bias [46], which limits generalisability of the findings.

In summary, this study highlights the interplay between adiposity, ageing, and disease risk and emphasises the limitations of BMI alone as a marker of cardiometabolic health. Our findings specifically demonstrate that VAT and ectopic fat depots are key contributors to the risk of T2DM and hyperlipidaemia, beyond what is captured by BMI alone. While the association with hypertension appeared to be mainly explained by BMI, associations with myocardial and cerebral infarction were less clear, likely due to limited incident cases, but these traits may still contribute through shared mechanisms of adipose tissue dysfunction. The findings underscore the importance of considering fat distribution and organ-specific fat accumulation when assessing risk for metabolic diseases, particularly type 2 diabetes and hyperlipidaemia. The cost and logistical demands of MRI limit its implementation in routine clinical risk assessment. Future clinical translation may therefore depend on the development of more scalable tools or biological markers for assessment of body fat distribution and ectopic fat deposition. Such approaches could improve risk assessment beyond BMI while preserving the practicality required for everyday clinical practice.

## Supplementary information

Supplementary Information

Supplementary Data S1

Supplementary Data S2

Supplementary Data S3

Supplementary Data S4

Supplementary Data S5

## Data Availability

The data for this study is available for researchers from the UK Biobank resource (https://www.ukbiobank.ac.uk/) and can be assessed by an application to the UK biobank. GWAS summary statistics for MRI-derived exposures are deposited in Zenodo [https://doi.org/10.5281/zenodo.19590199].

## Code availability

Code have been deposited in GitHub [https://github.com/AJResearchGroup/MRI_adiposity_Cardiometabolic].
