# Supplementary material for: Age-related changes in adiposity and cardiometabolic disease risk: a longitudinal and prospective study in the UK Biobank
Source: Int J Obes (Lond). 2026 Jul 15;50(10):2185–94. doi: 10.1038/s41366-026-02168-2 (PMC13722588; doi:10.1038/s41366-026-02168-2)
Supplement: Supplementary file 1 [file 41366_2026_2168_MOESM1_ESM.pdf]

Supplementary Information

Age-related changes in adiposity and cardiometabolic disease risk: A longitudinal and prospective study in the UK Biobank

Table of Contents

Supplementary Methods 3

UK Biobank cohort 3

MRI-based phenotypes 3

Age-related differences/changes in adiposity traits 3

Cox-proportional hazards modelling 4

Genetic instruments for Mendelian Randomization 5

Mediation analyses using Multivariable Mendelian Randomization 5

Supplementary Information References 6

Supplementary Figures 7

Figure S1. Rate of change per year of age. 7
Figure S2. The rates of change in anthropometric and abdominal adiposity measurements with age (cross-sectional data). 8
Figure S3. The rates of change in ectopic fat deposition and organ volume with age (cross-sectional data). 9
Figure S4. The rates of change in adiposity traits with age (cross-sectional and longitudinal). 10
Figure S5. The rates of change in upper body adiposity with age (cross-sectional and longitudinal). 11
Figure S6. The rates of change in organ fat composition and volume with age (cross-sectional and longitudinal). 12
Figure S7. The rates of change in thigh muscle fat infiltration and fat-free muscle volume with age (cross-sectional and longitudinal). 13
Figure S8. Changes in pancreatic- and liver fat amounts and volumes with age (cross-sectional and longitudinal). 14
Figure S9. Changes in anthropometric measures of adiposity with age in other ethnicities. 15
Figure S10. Changes in abdominal adiposity with age in other ethnicities. 16
Figure S11. Changes in pancreatic and liver composition and volume with age other ethnicities. 17
Figure S12. Changes in thigh muscle fat infiltration and fat-free muscle volume with age in other ethnicities. 18

Figure S13. Scaled Schoenfeld residuals plot of the effect of VAT on the rate of incident diabetes in females (Beta(t)). 19
Figure S14. Mendelian randomization estimates of the causal effects of adiposity traits on type 2 diabetes and hyperlipidaemia. 20

Supplementary Tables 21

Table S1: Details of GWAS included in Mendelian randomization analyses. 21
Table S2. Characteristics of UK Biobank participants at initial assessment (instance 0) by sex and MRI participation status. 22
Table S3. Self-reported ethnic background of UK Biobank participants at the initial MRI imaging session. 23
Table S4. Stepwise Cox proportional hazards regression to determine informative traits for the rate of incident type 2 diabetes. 23
Table S5. Stepwise Cox proportional hazards regression to determine informative traits for the rate of incident hyperlipidemia. 23
Table S6. Difference-in-coefficients analyses of adiposity traits on BMI-associated type 2 diabetes risk in female and male UK Biobank participants. 24
Table S7. Difference-in-coefficients analyses of adiposity traits on BMI-associated hyperlipidaemia risk in female and male UK Biobank participants. 25

Supplementary Methods

UK Biobank cohort

The UK Biobank (UKB) is a population-based cohort study involving approximately 500,000 UK residents aged 37–73 years at recruitment between 2006 and 2010. Invitations were sent to 9.2 million UK residents, with ~500,000 participants assessed at 22 centers across the UK between 2006 and 2010. Participants provided biological samples, anthropometric data, and information on sociodemographic and lifestyle factors through a touchscreen questionnaire and interview. The UKB is linked to national health registers, offering data on cancer diagnoses, hospital inpatient diagnoses, primary care, and causes of death, enabling prospective disease studies. Anthropometric measurements, including height, weight, waist and hip circumferences, were taken at the assessment visits, including the recruitment visit (2006-2010) but also the imaging visits that started in 2014.

MRI-based phenotypes

MRI scans were performed using a Siemens Area 1.5 Tesla MAGNETOM Area scanner (Siemens Healthineers, Erlangen, Germany) [1]. Participants underwent a Dixon imaging program that covered from the neck to the knees to capture images of abdominal adipose tissue. The LiverMultiScan protocol (Perspectum Diagnostics, Oxford, UK) was used as a multiparametric MRI method to produce quantitative measures of liver health: corrected T1 (cT1), which reflects fibro-inflammatory activity; proton density fat fraction (PDFF), which measures liver fat content; and liver iron concentration (LIC), which measures hepatic iron load. Additional imaging included the single breath-hold cardiac-gated Modified Look-Locker Inversion Recovery sequence (shMOLLI) protocol for T1 mapping of the liver, a multi-echo sequence for quantification of liver iron and PDFF, a 3D volumetric interpolated breath-hold examination (VIBE) sequence for volumetric evaluation of the pancreas, and a multi-echo sequence to determine pancreatic iron and PDFFs [1].

Age-related differences/changes in adiposity traits

We assessed age-related differences/changes in adiposity in two UK Biobank datasets: cross-sectional baseline MRI measurements and longitudinal repeat MRI measurements. Participants' ages and time between imaging sessions were calculated using birth years and assessment dates (data field 33 and 53). While a much larger sample size was available for the cross-sectional baseline samples, there is a risk that participants with different ages at recruitment exhibit differences in adiposity that are not due to age, but rather to unmeasured time trends that may bias the estimated effects of age, such as socioeconomic and food-related exposures earlier in life. In the longitudinal analysis, we computed the annual within-person rate of change for each participant as (second imaging measure – first imaging measure)/time between scans (years) and treated this rate as constant over the interval. To summarise by age, for each year of age a we averaged these interval rates across all participants whose scan interval included age a. These sex-stratified means trace the average annual change by age (units: trait/year). A 95% confidence interval (CI) that excluded zero indicated a positive or negative yearly rate of change (Figure S1).

Cross-sectional data

To quantify and compare the cross-sectional and longitudinal data, we model the cross-sectional trajectory with a quadratic function of age. Specifically,

$$Trait(a) = \beta_0 + \beta_1 \cdot a + \beta_2 \cdot a^2 \qquad (Eq.\ 1),$$

where a is age (years) and Trait is the adiposity measure. Here, β_0 is the intercept (Trait at a = 0), and β_1 and β_2 capture the linear and quadratic age effects. We estimate β_0–β_2 by polynomial regression and use F-tests on β_1 and β_2 to assess whether age is associated with the trait, and whether the association is non-linear ($\beta_2 \neq 0$). The quadratic form permits non-linear age effects, for example a rise at younger ages followed by a plateau and a subsequent decline at older ages; this pattern corresponds to a rate of change that declines with age.

Uncertainty in the inferred plateau ages was quantified using parametric Bootstrap simulations (10,000,000 replicates). Specifically, the model coefficient estimators were assumed to be normally distributed. New beta estimates for age and age^2 were sampled using a multivariate normal distribution with means being the estimated beta effects from data, while the variance-covariance matrix were constructed from the corresponding standard-error estimates and assuming perfect anti-correlation between the two beta estimators. The turning point was then recalculated for each pair of sampled beta estimates across simulations. From the generated distribution of turning points, we report the median and corresponding confidence intervals for the plateau age estimates.

Longitudinal model

For the longitudinal data, we model the annual within-person rate of change as a linear function of age:

$$r_i = \gamma_0 + \gamma_1 a_i + \varepsilon_i \qquad (Eq.\ 2)$$

where $r_i = \Delta Trait_i / \Delta time_i$ (units per year), a_i is age (years), γ_0 is the intercept, and γ_1 is the slope. We estimate γ_0 and γ_1 by linear regression and test $H_0\colon \gamma_1 = 0$ to assess whether the rate of change depends on age (two-sided t-test with n-2 df where n is number of individuals; equivalent F-test with (1,n-2) df).

Linking cross-sectional and longitudinal results

Eq. 2 (the derivative of the cross-sectional model) has the same form as Eq. 3 (the longitudinal model of rates). Accordingly, γ_0 corresponds to β_1, and γ_1 corresponds to $2\beta_2$. We compared γ_1 with $2\beta_2$ (and, where relevant, γ_0 with β_1) using two-sided t-tests to assess agreement between designs.

Cox-proportional hazards modelling

All models were also adjusted for the first 15 genetic principal components, derived from genome-wide genotype data as provided by UK Biobank, to account for population stratification by capturing major axes of genetic ancestry. We aimed to keep the model parsimonious, using a limited set of well-validated covariates, as in clinically established risk prediction tools. This approach reduces the risk of overadjustment and ensures consistency with models applicable in real-world settings, while additional factors, though potentially relevant, are either not included in clinical scores or may be prone to measurement error (e.g. self-reported diet and physical activity). The 15 genetic principal components, derived

from genome-wide genetic data, capture population structure and ancestry differences, helping to control for potential confounding due to genetic background.

Genetic instruments for Mendelian Randomization

We conducted two-sample MR analyses to confirm the effects of BMI and other adiposity traits on disease outcomes, particularly where adiposity traits showed BMI-independent effects. Genetic instruments for 12 adiposity traits were identified from UK Biobank GWAS using Plink v2 [2]. Traits were normalized by rank-based inverse normal transformation separately for males and females. Linear regression models tested the effects of single-nucleotide polymorphisms (SNPs) on these traits, adjusting for sex, age, genotyping batch, and principal components. Publicly available GWAS datasets for BMI, waist circumference, and WHR were also used to increase the number of independent instruments (Table S1). SNP effect estimates were extracted from the GIANT summary statistics [3,4]. SNPs that were strongly associated with the exposures ($p < 5*10^{-8}$), and which were independent (pairwise $R^2 < 0.001$ or located > 5000 kb apart) were selected as instrumental variables. The effect estimates for instrumental variables for the selected disease outcomes (T2DM, hyperlipidaemia and hypertension) were also selected from publicly available datasets (Table S1) from DIAGRAM or FINNGEN [5,6].

GWAS summary-level data were harmonized to align genetic instruments across datasets. Palindromic variants were removed using allele frequencies, and SNPs with major allele frequencies above 45% were excluded due to strand orientation issues. Steiger filtering was applied to all genetic instruments to ensure that each SNP explained more variance in the exposure than in the outcome, thereby reducing the risk of reverse causation and the F-statistic was used to assess the strength and validity of instrumental variables, with values below 10 indicating potential bias and necessitating further evaluation of their reliability [7]. The number of instrumental variables for traits and diseases ranged from seven to 101, with F-statistics spanning 34 to 367, which meets the recommended thresholds for robust MR analyses [8].

Mediation analyses using Multivariable Mendelian Randomization

Multivariable MR (MVMR) regression was used as an independent extension of the previous analyses to explore how other adiposity traits contribute to the associations between BMI-associated genetic instruments and disease risk. Analyses were performed with the 'MendelianRandomization' R-package [9]. MVMR is an extension of the Mendelian randomization framework that allows for the assessment of independent causal effects of multiple exposures on a single outcome. This method can also be used to assess the proportion of an effect that acts through a related exposure, i.e. analogous to traditional mediation analyses [10]. The genetic variants used in MVMR were combinations of IVs for each exposure, with SNP exclusion criteria consistent with univariable analyses. A two-sample conditional F-statistic was calculated to assess how well genetic variants predict each exposure, conditional on others in the MVMR model. Results were considered valid when the conditional F-statistic was >10.

Indirect effects were approximated using a difference-in-coefficients approach, calculated as the difference between the univariable MR estimate for BMI on the outcome and the corresponding MVMR estimate adjusted for adiposity traits. This reflects the extent to which the total effect of BMI may be attributable to these traits. Random-effects inverse-variance weighted models were used as the primary method. Sobel’s tests were applied to assess

whether the attenuation of the BMI–outcome association was statistically significant, with two-sided p-values reported.

Supplementary Information References

1 Littlejohns TJ, Holliday J, Gibson LM, Garratt S, Oesingmann N, Alfaro-Almagro F *et al.* The UK Biobank imaging enhancement of 100,000 participants: rationale, data collection, management and future directions. *Nat Commun* 2020; **11**: 2624.

2 Chang CC, Chow CC, Tellier LCAM, Vattikuti S, Purcell SM, Lee JJ. Second-generation PLINK: Rising to the challenge of larger and richer datasets. *GigaScience* 2015; **4**. doi:10.1186/s13742-015-0047-8.

3 Turcot V, Lu Y, Highland HM, Schurmann C, Justice AE, Fine RS *et al.* Protein-altering variants associated with body mass index implicate pathways that control energy intake and expenditure in obesity. *Nat Genet* 2018; **50**: 26–41.

4 Shungin D, Winkler TW, Croteau-Chonka DC, Ferreira T, Locke AE, Mägi R *et al.* New genetic loci link adipose and insulin biology to body fat distribution. *Nature* 2015; **518**: 187–196.

5 Mahajan A, Taliun D, Thurner M, Robertson NR, Torres JM, Rayner NW *et al.* Fine-mapping type 2 diabetes loci to single-variant resolution using high-density imputation and islet-specific epigenome maps. *Nat Genet* 2018; **50**: 1505–1513.

6 Kurki MI, Karjalainen J, Palta P, Sipilä TP, Kristiansson K, Donner KM *et al.* FinnGen provides genetic insights from a well-phenotyped isolated population. *Nature* 2023; **613**: 508–518.

7 Sanderson E, Spiller W, Bowden J. Testing and correcting for weak and pleiotropic instruments in two-sample multivariable Mendelian randomization. *Stat Med* 2021; **40**: 5434–5452.

8 Sanderson E, Glymour MM, Holmes MV, Kang H, Morrison J, Munafò MR *et al.* Mendelian randomization. *Nat Rev Methods Primer* 2022; **2**: 6.

9 Yavorska O, Staley J. MendelianRandomization. 2023.https://cran.r-project.org/web/packages/MendelianRandomization/index.html.

10 Sanderson E. Multivariable Mendelian Randomization and Mediation. *Cold Spring Harb Perspect Med* 2021; **11**: a038984.

Supplementary Figures

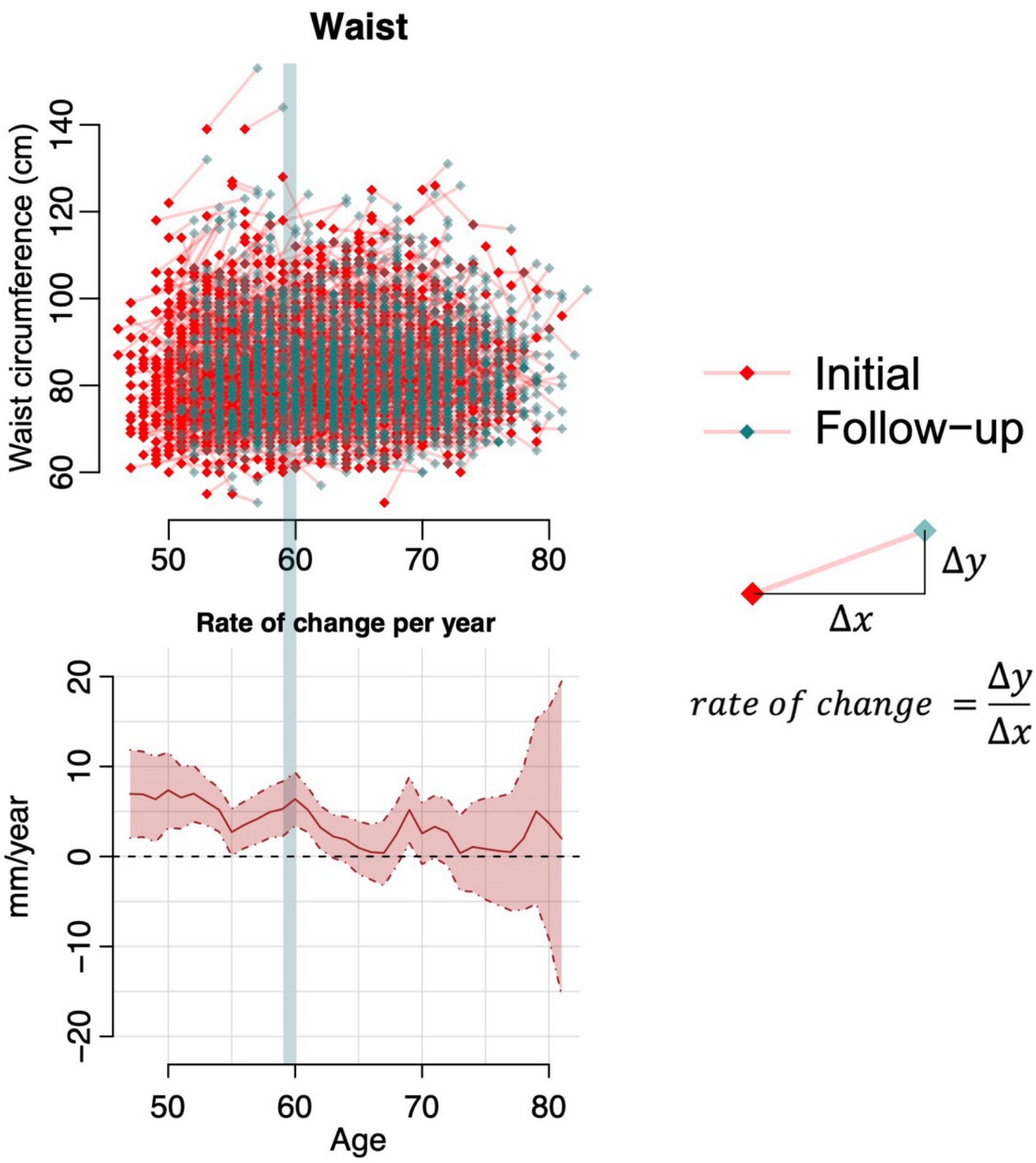

Figure S1. Rate of change per year of age.
For each participant with two MRIs, we computed the within-person annual rate (follow-up−baseline)/Δt and assumed it constant over the interval. For each age, we averaged these rates across all participants whose scan-interval includes that age. The blue vertical band marks the age slice (here ~59–60 years) used for this averaging; each overlapping participant contributes the same interval rate to that slice. The example panel shows the change in waist circumference with age in females.

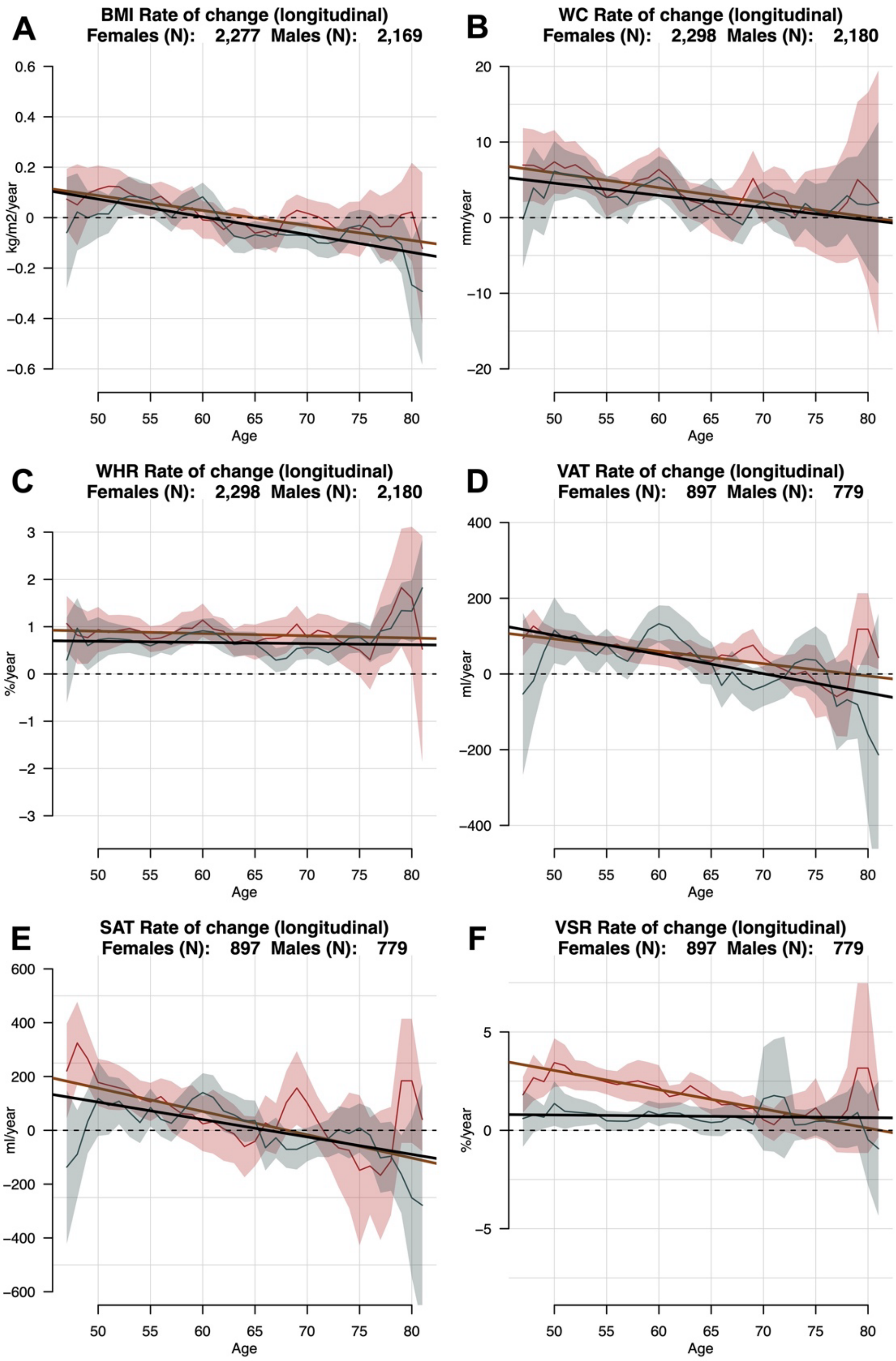

Figure S2. The rates of change in anthropometric and abdominal adiposity measurements with age (cross-sectional data).

The average rate of change, as assessed from longitudinal data, are presented for females (red) and males (green). Shaded regions denote 95% confidence intervals. The age at which the lines cross the x-axis corresponds to the plateau—the age when a trait changes from increasing to decreasing in value. Abbreviations: BMI = Body mass index, WC = waist circumference, WHR = Waist-to-hip ratio, VAT = Visceral adipose tissue volume, SAT = Subcutaneous adipose tissue volume, VSR = Visceral-to-subcutaneous adipose tissue ratio, i.e. the proportion of visceral- to subcutaneous adipose tissue volume.

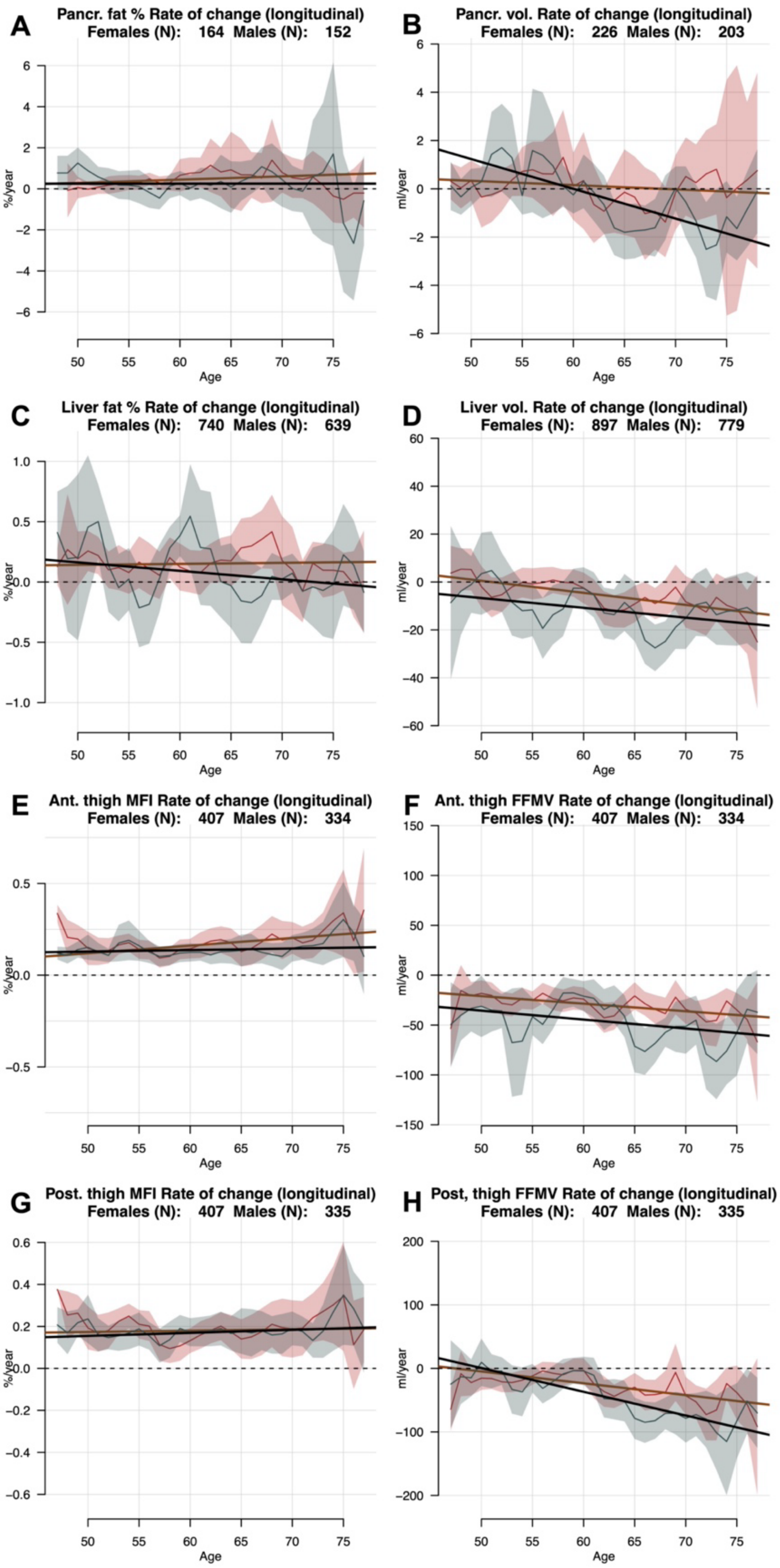

Figure S3. The rates of change in ectopic fat deposition and organ volume with age (cross-sectional data). Data are presented separately for females (red) and males (green). The average rates of change, as assessed from longitudinal data, are presented. Shaded regions denote 95% confidence intervals. The age at which the lines cross the x-axis corresponds to the plateau—the age when a trait changes from increasing to decreasing in value. Abbreviations: Pancr. = Pancreas, Vol. = volume, Ant. = Anterior, Post. Posterior, MFI = Muscle fat infiltration. FFMV = Fat free muscle volume.

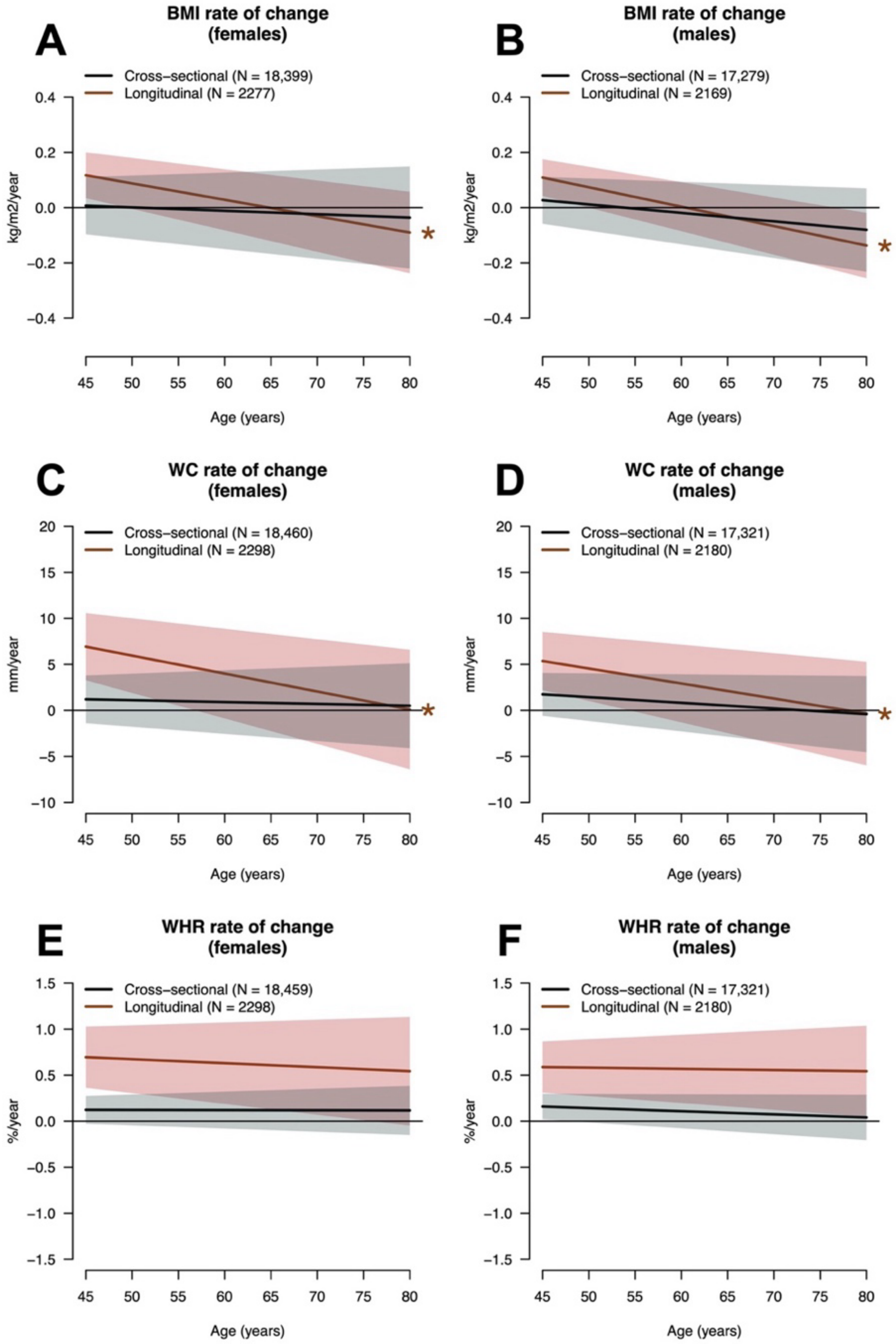

Figure S4. The rates of change in adiposity traits with age (cross-sectional and longitudinal).
For the longitudinal data (brown), the line was estimated with a first-degree polynomial function: $y \sim \beta*x + M$, where y is the rate of change in the trait per year, x is age in years, M is the intercept and β is the regression coefficient = the slope of the line). For the cross-sectional data (black) a second-degree polynomial function ($y \sim \beta_1*x + \beta_2*x^2 + M$) was fitted where y is the trait values at age x. The derivative of the second-degree polynomial then represents the change in rate with age for the cross-sectional data. Shaded areas denote 95% confidence intervals of the fitted curves. Asterisks denote a significant deviation of the slopes from zero, after Bonferroni correction for adjusting for multiple testing (P < 8.9E-4, which corresponds to 56 independent tests: 14 traits, longitudinal and cross-sectional data, in female and male participants), which indicates that the rate is dependent on age. Asterisks are coloured according to the data which they represent: longitudinal (brown) or cross-sectional (black). The age at which the lines cross the x-axis corresponds to the plateau, i.e. the age when a trait stops increasing and begins to decrease in value. BMI = Body mass index, WC = waist circumference, WHR = waist-to-hip ratio.

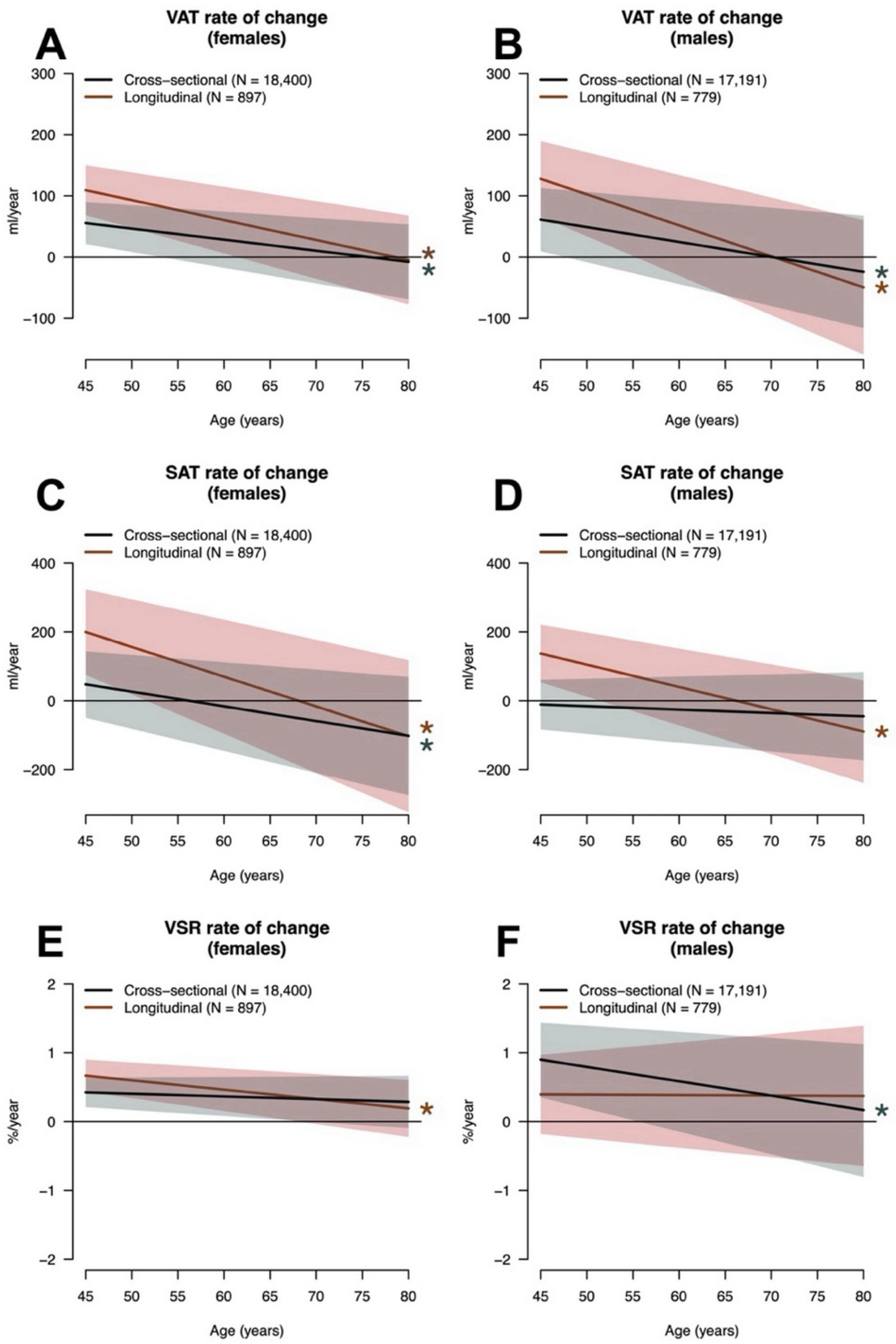

Figure S5. The rates of change in upper body adiposity with age (cross-sectional and longitudinal). For the longitudinal data (brown), the line was estimated first-degree polynomial function: y ~ β*x + M, where y is the rate of change in the trait per year, x is age in years, M is the intercept and β is the regression coefficient = the slope of the line). For the cross-sectional data (black) a second-degree polynomial function (y ~ β1*x + β2*x2 + M) was fitted where y is the trait values at age x. The derivative of the second-degree polynomial then represents the change in rate with age for the cross-sectional data. Shaded areas denote 95% confidence intervals of the fitted curves. Asterisks denote a significant deviation of the slopes from zero, after Bonferroni correction for adjusting for multiple testing (P < 8.9E-4, which corresponds to 56 independent tests: 14 traits, longitudinal and cross-sectional data, in female and male participants), which indicates that the rate is dependent on age. Asterisks are coloured according to the data which they represent: longitudinal (brown) or cross-sectional (black). The age at which the lines cross the x-axis corresponds to the plateau—the age when a trait changes from increasing to decreasing in value. Asterisks are coloured according to the data which they represent: longitudinal (brown) or cross-sectional (black). Abbreviations: VAT = Visceral adipose tissue volume, SAT = subcutaneous adipose tissue volume, VSR = Visceral-to-subcutaneous tissue volume ratio.

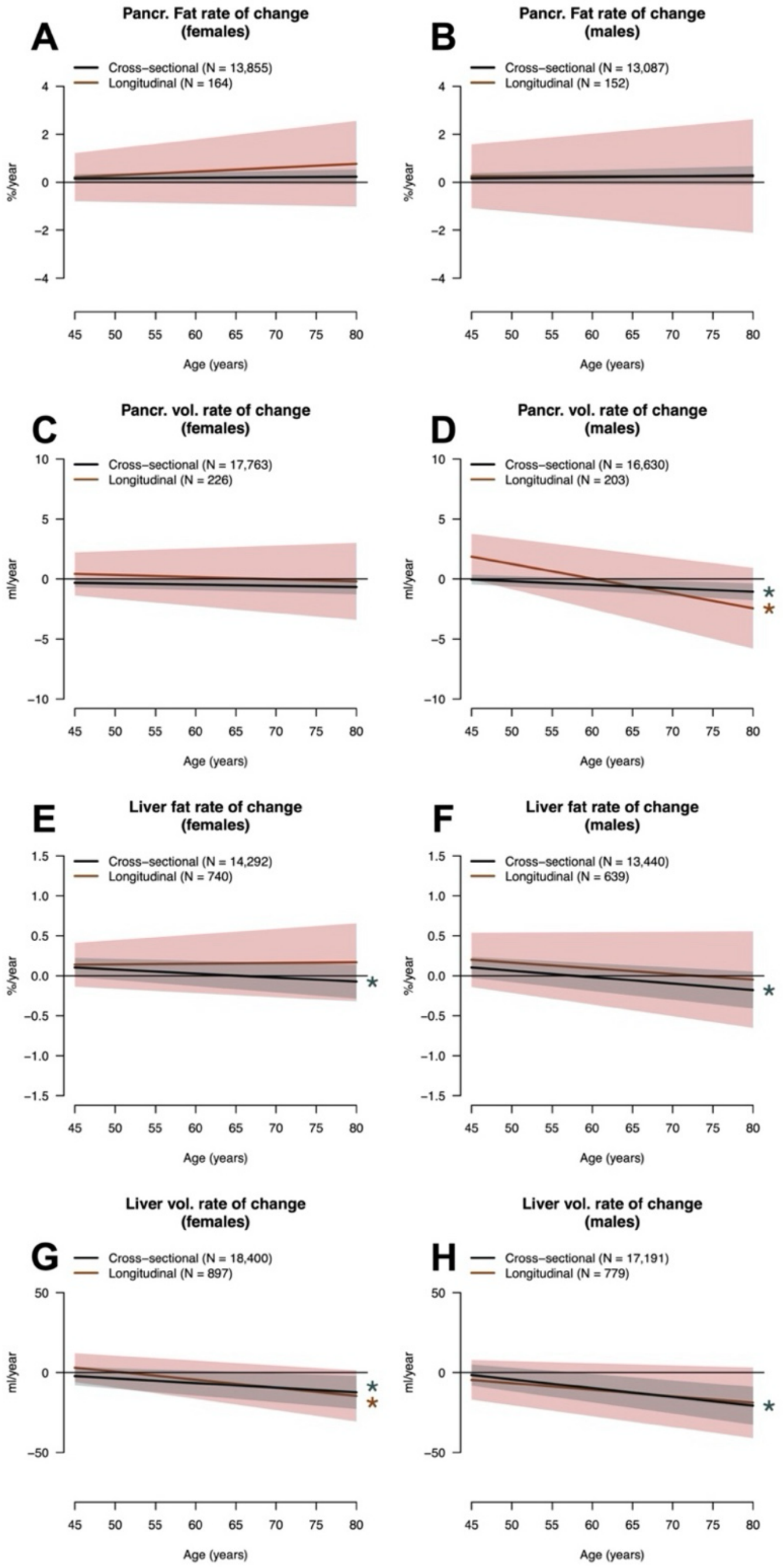

Figure S6. The rates of change in organ fat composition and volume with age (cross-sectional and longitudinal).

For the longitudinal data (brown), the line was estimated first-degree polynomial function: $y \sim \beta * x + M$, where y is the rate of change in the trait per year, x is age in years, M is the intercept and β is the regression coefficient = the slope of the line). For the cross-sectional data (black) a second-degree polynomial function ($y \sim \beta1*x + \beta2*x2 + M$) was fitted where y is the trait values at age x. The derivative of the second-degree polynomial then represents the change in rate with age for the cross-sectional data. Shaded areas denote 95% confidence intervals of the fitted curves. Asterisks denote a significant deviation of the slopes from zero, after Bonferroni correction for adjusting for multiple testing ($P < 8.9E-4$, which corresponds to 56 independent tests: 14 traits, longitudinal and cross-sectional data, in female and male participants), which indicates that the rate is dependent on age. Asterisks are coloured according to the data which they represent: longitudinal (brown) or cross-sectional (black). The age at which the lines cross the x-axis corresponds to the plateau—the age when a trait changes from increasing to decreasing in value. Asterisks are coloured according to the data which they represent: longitudinal (brown) or cross-sectional (black). Pancr. = pancreas, vol. = volume.

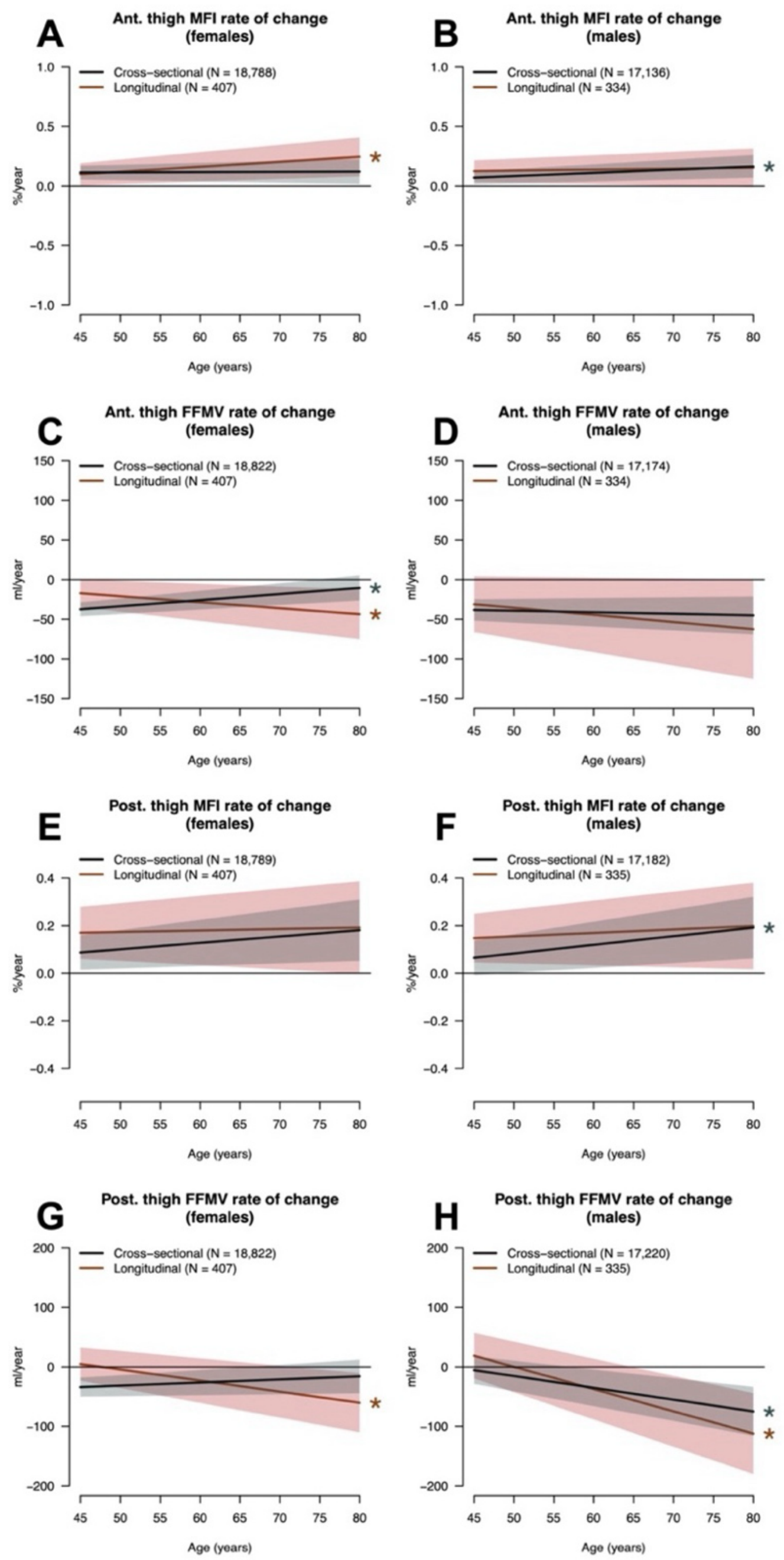

Figure S7. The rates of change in thigh muscle fat infiltration and fat-free muscle volume with age (cross-sectional and longitudinal).

For the longitudinal data (brown), the line was estimated first-degree polynomial function: $y \sim \beta * x + M$, where y is the rate of change in the trait per year, x is age in years, M is the intercept and β is the regression coefficient = the slope of the line). For the cross-sectional data (black) a second-degree polynomial function ($y \sim \beta_1 * x + \beta_2 * x^2 + M$) was fitted where y is the trait values at age x. The derivative of the second-degree polynomial then represents the change in rate with age for the cross-sectional data. Shaded areas denote 95% confidence intervals of the fitted curves. Asterisks denote a significant deviation of the slopes from zero, after Bonferroni correction for adjusting for multiple testing (P < 8.9E-4, which corresponds to 56 independent tests: 14 traits, longitudinal and cross-sectional data, in female and male participants), which indicates that the rate is dependent on age. Asterisks are coloured according to the data which they represent: longitudinal (brown) or cross-sectional (black). The age at which the lines cross the x-axis corresponds to the plateau—the age when a trait changes from increasing to decreasing in value. Asterisks are coloured according to the data which they represent: longitudinal (brown) or cross-sectional (black). Pancr. = pancreas, vol. = volume. Post. = Posterior, Ant. = Anterior, MFI = Muscle fat infiltration, FFMV = Fat-free muscle volume.

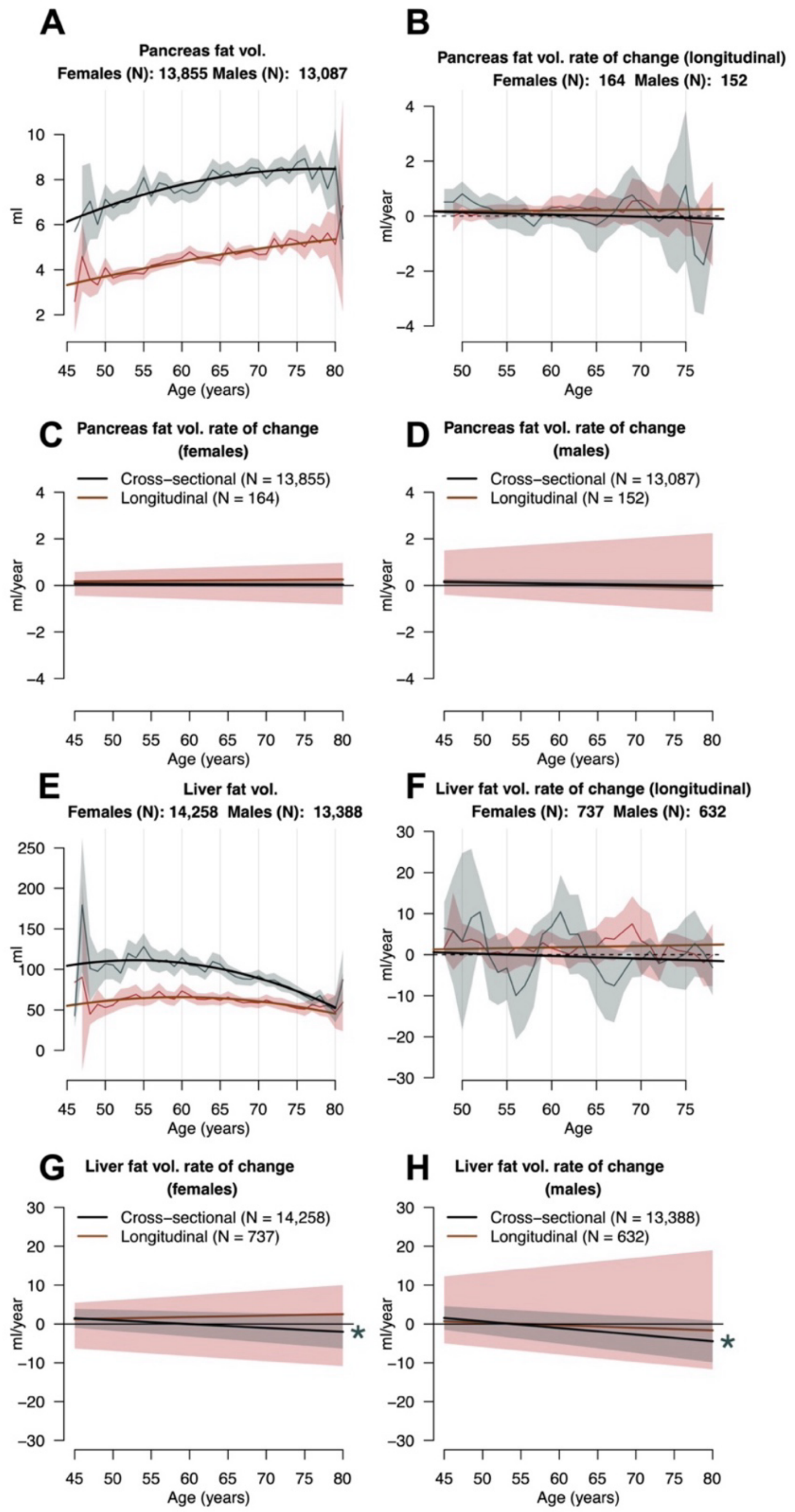

Figure S8. Changes in pancreatic- and liver fat amounts and volumes with age (cross-sectional and longitudinal).

Cross sectional averages at initial imaging for each year of age with 95% confidence intervals (shaded areas) are presented (A +E). Second degree polynomials were fitted to the data using polynomial regression modelling in R (solid lines). (B+F) The average rates of change, as assessed from longitudinal data. Shaded regions denote 95% confidence intervals. (C+D, G+H) The rates of change in adiposity traits were estimated by linear regression in the longitudinal data and by fitting a second-degree polynomial in the cross-sectional data (black). The derivative of the second-degree polynomial is used to illustrate the change in rate with age. Shaded areas denote 95% confidence intervals of the fitted curve. Asterisks denote a significant deviation of the slopes of β and the derivative of β_2 from zero, after Bonferroni correction for adjusting for multiple testing ($P < 8.9E-4$, which corresponds to 56 independent tests: 14 traits, longitudinal and cross-sectional data, in female and male participants), which indicates that the rate of change is dependent on age.

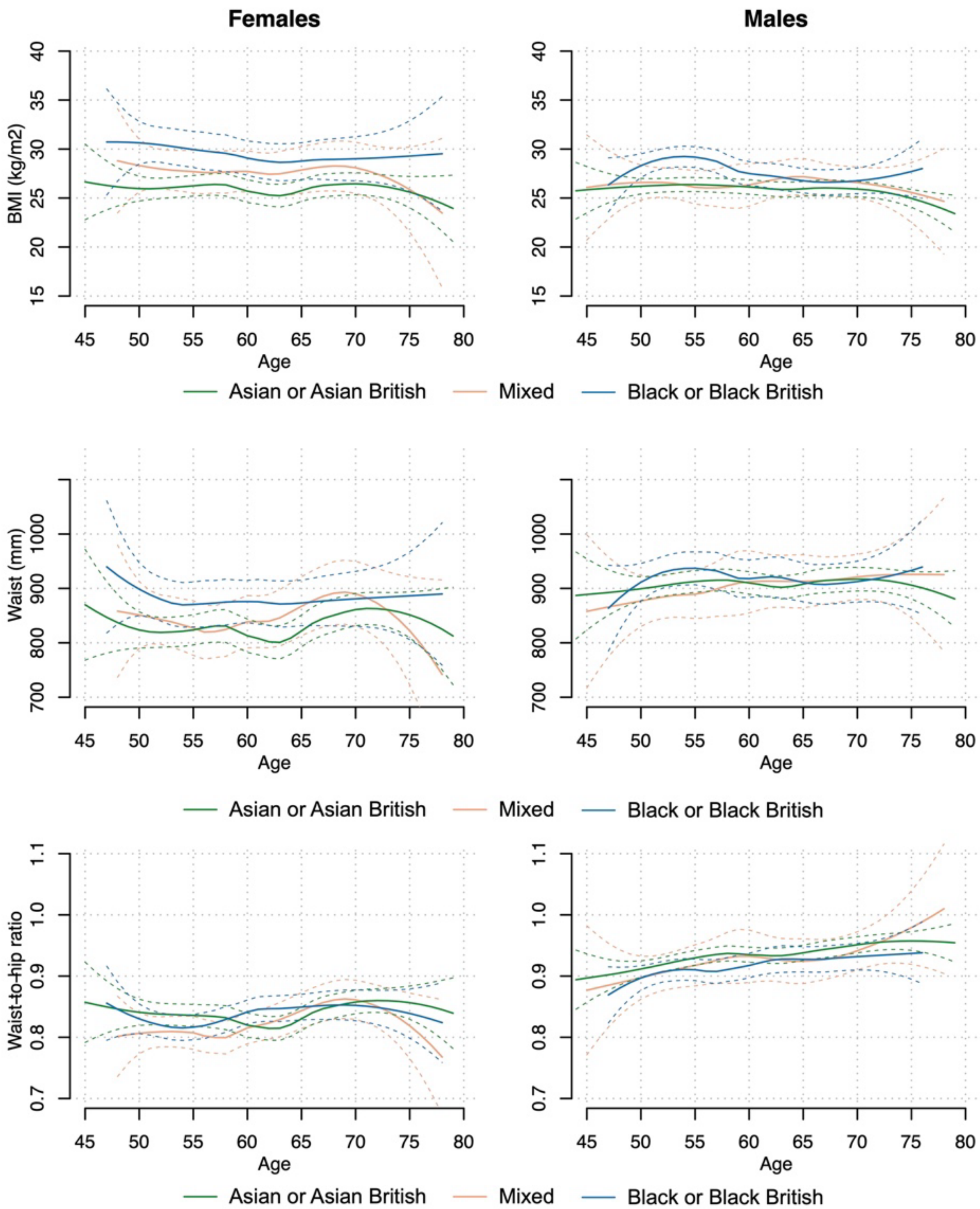

Figure S9. Changes in anthropometric measures of adiposity with age in other ethnicities.
Data are presented separately for females (left column) and males (right column). Solid lines represent moving averages generated by locally estimated scatterplot smoothing (LOESS) using the 'loess' function in R using 2nd degree polynomials with the smoothing parameter set to 0.75. Dashed lines represent the upper and lower limits of 95% confidence intervals for each ethnic group (dashed lines).

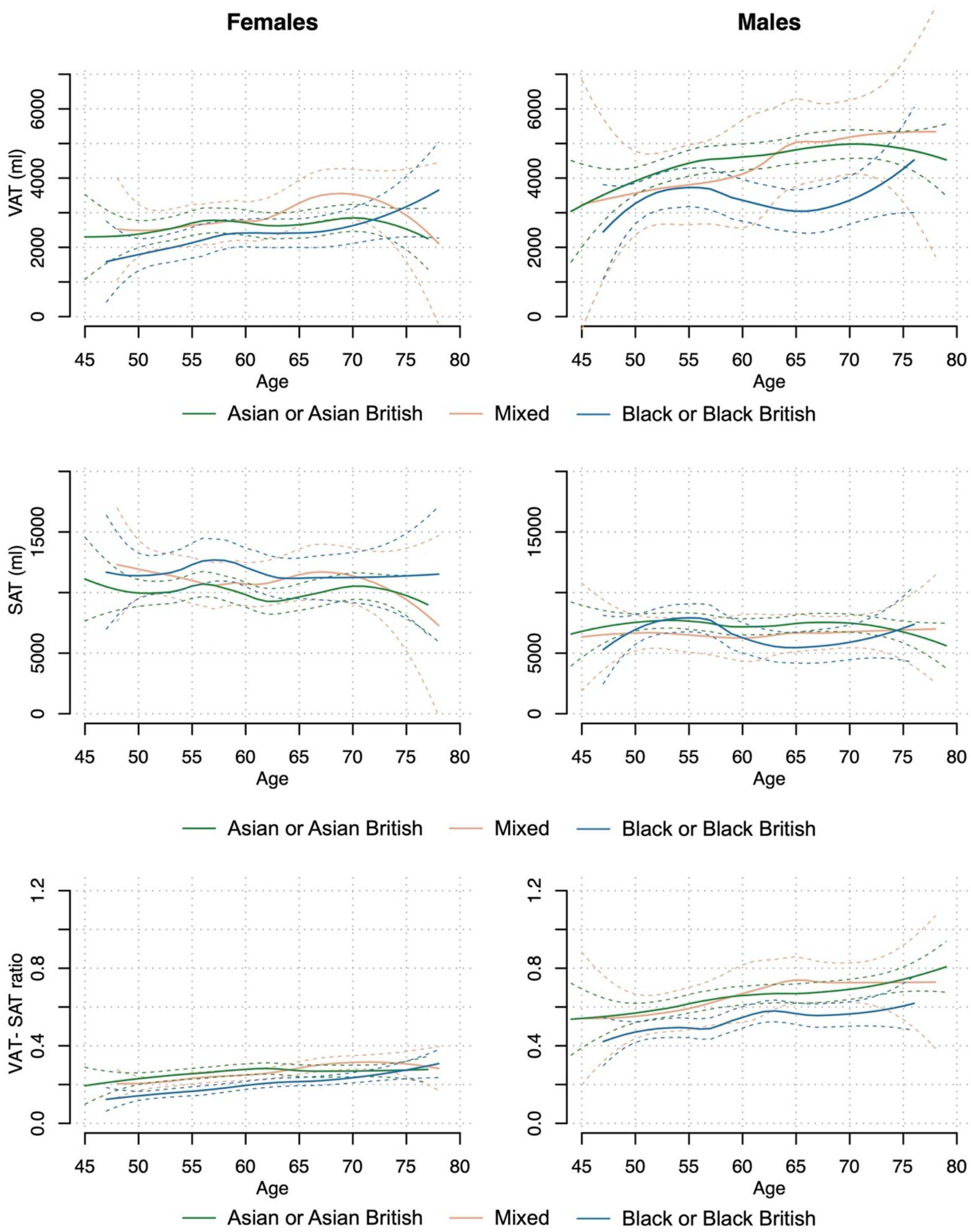

Figure S10. Changes in abdominal adiposity with age in other ethnicities.
Data are presented separately for females (left column) and males (right column). Solid lines represent moving averages generated by locally estimated scatterplot smoothing (LOESS) using the 'loess' function in R using 2nd degree polynomials with the smoothing parameter set to 0.75. Dashed lines represent the upper and lower limits of 95% confidence intervals for each ethnic group (dashed lines).

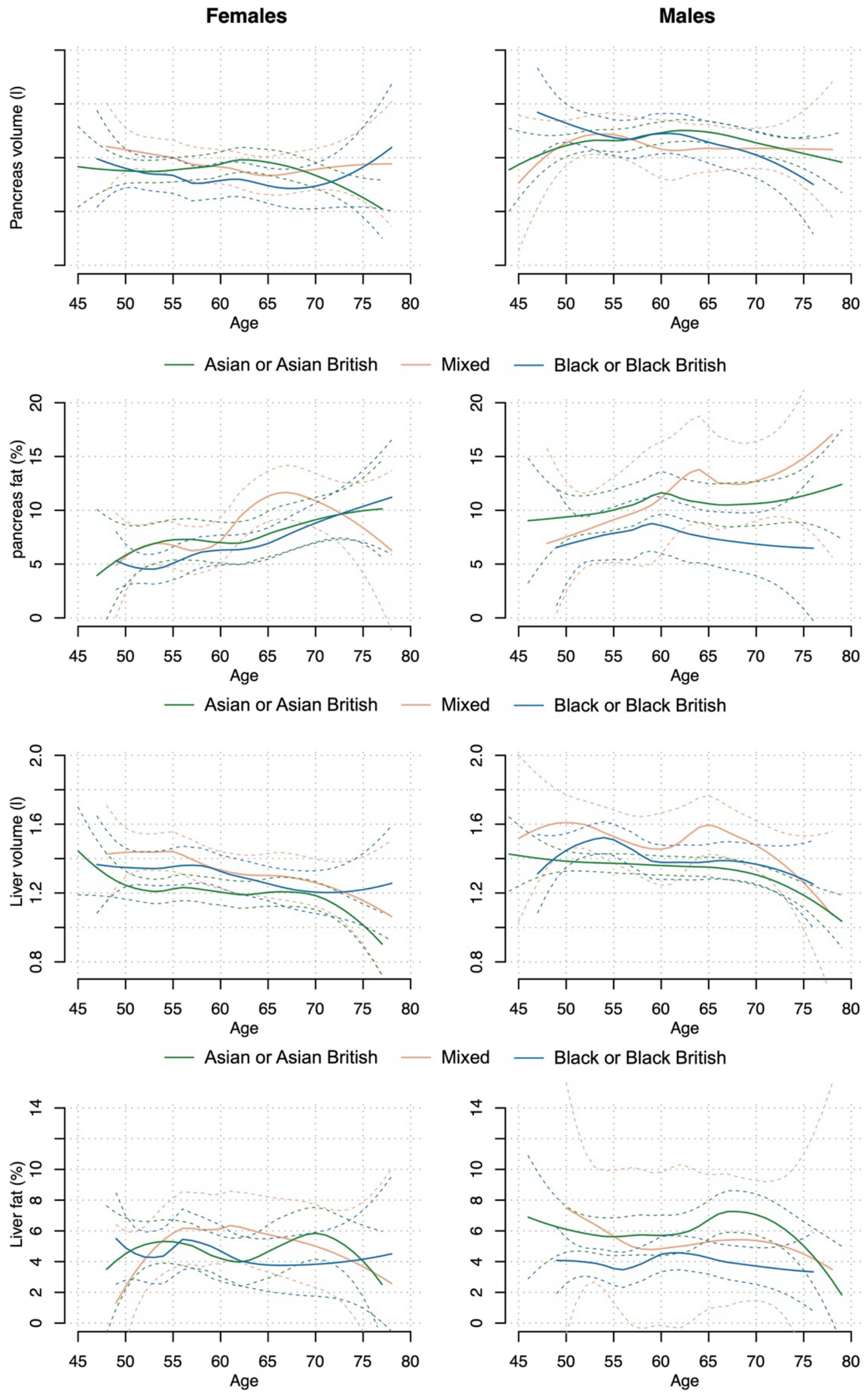

Figure S11. Changes in pancreatic and liver composition and volume with age other ethnicities.
Data are presented separately for females (left column) and males (right column). Solid lines represent moving averages generated by locally estimated scatterplot smoothing (LOESS) using the 'loess' function in R using 2nd degree polynomials with the smoothing parameter set to 0.75. Dashed lines represent the upper and lower limits of 95% confidence intervals for each ethnic group (dashed lines).

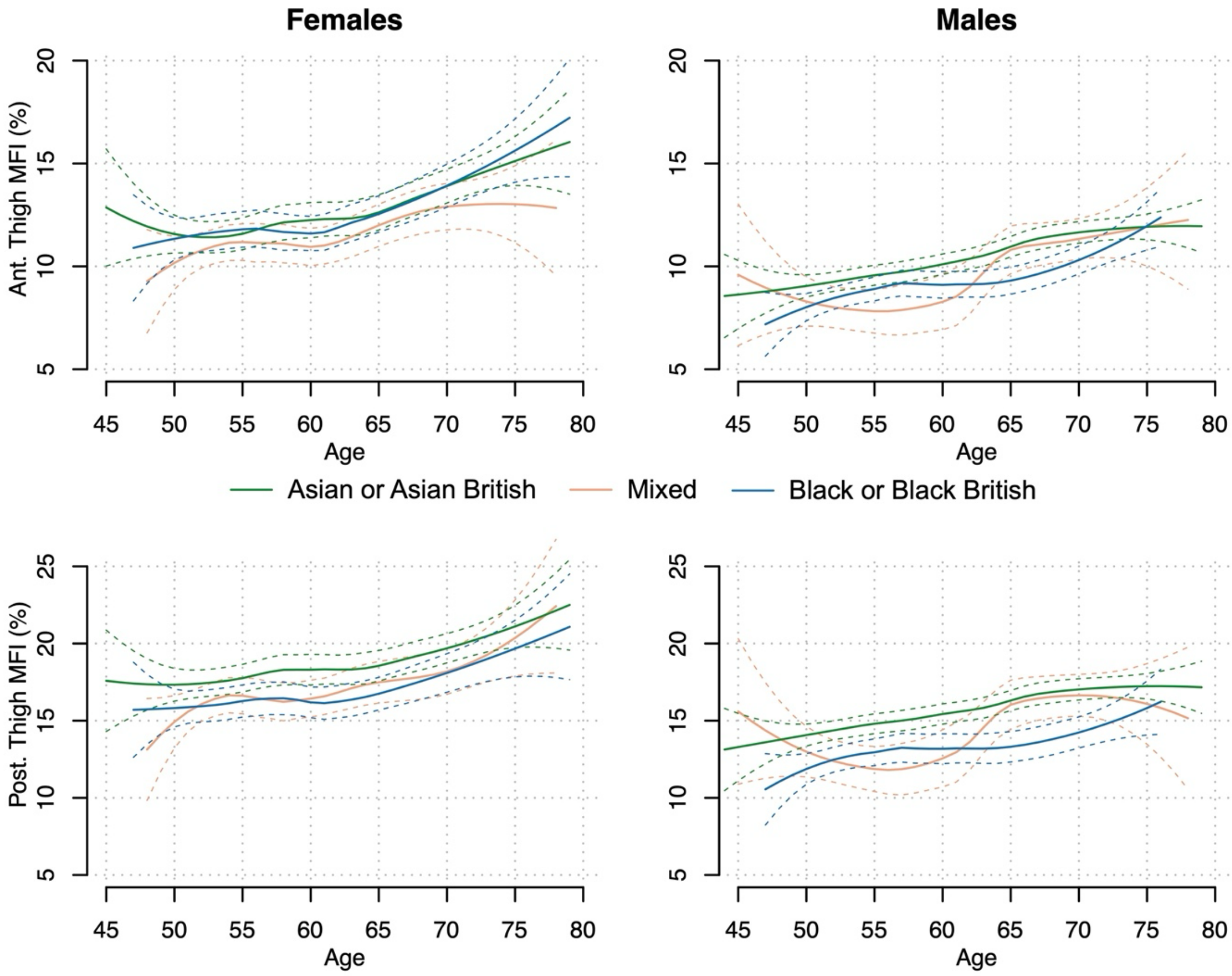

Figure S12. Changes in thigh muscle fat infiltration and fat-free muscle volume with age in other ethnicities. Data are presented separately for females (left column) and males (right column). Solid lines represent moving averages generated by locally estimated scatterplot smoothing (LOESS) using the 'loess' function in R using 2nd degree polynomials with the smoothing parameter set to 0.75. Dashed lines represent the upper and lower limits of 95% confidence intervals for each ethnic group (dashed lines).

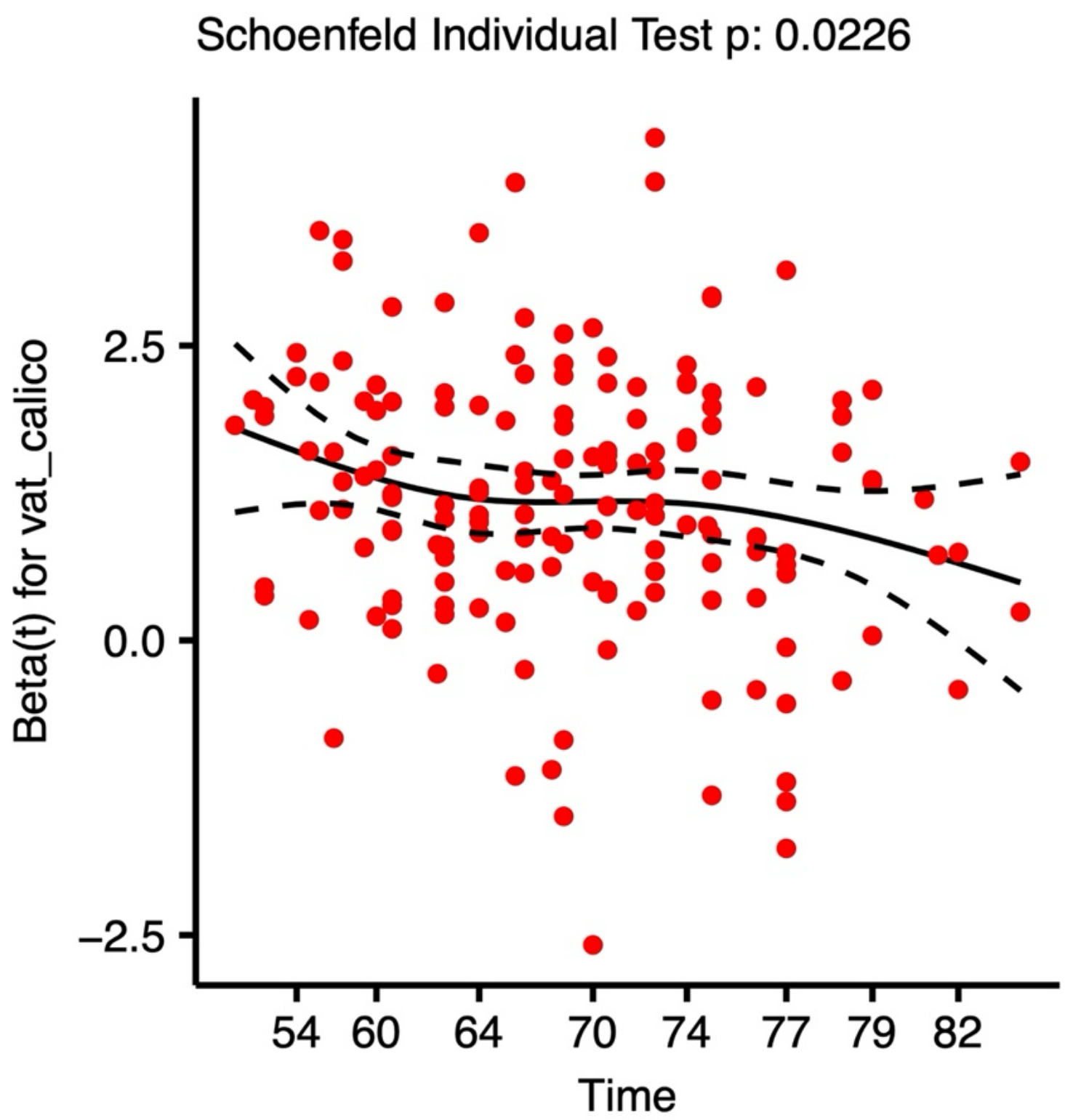

Figure S13. Scaled Schoenfeld residuals plot of the effect of VAT on the rate of incident diabetes in females (Beta(t)).

The black solid line is a smooth curve fit of the Schoenfeld residuals and dashed lines represent confidence bands at two standard errors. The P-value represents the results from a score test for deviance of the slope from zero and P < 0.05 is considered significant. Deviance from the proportionality assumption can thus be argued for this case. Likely, this is due to a diminishing association of VAT with the incident diabetes with older age in female UK Biobank participants.

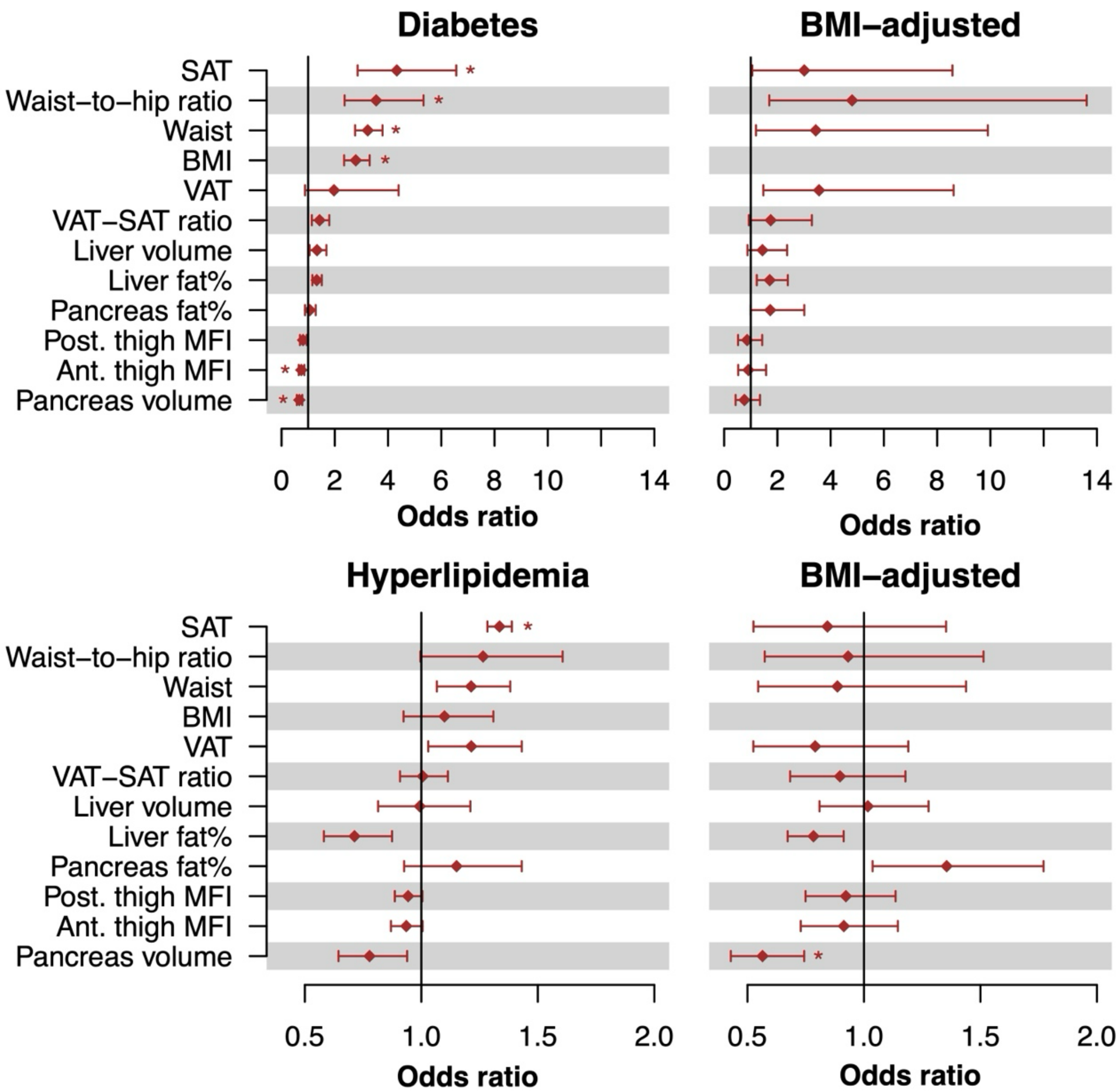

Figure S14. Mendelian randomization estimates of the causal effects of adiposity traits on type 2 diabetes and hyperlipidaemia.

The left panel shows results from univariable Mendelian randomization (MR), and the right panel shows results from multivariable MR (BMI-adjusted). Both panels display findings from the random-effects inverse-variance weighted (IVW) method. Odds ratios are presented with 95% confidence intervals. Asterisks denote statistical significance after Bonferroni correction (P<0.05/48). Abbreviations: VAT = visceral adipose tissue, SAT = subcutaneous adipose tissue, BMI = body mass index, MFI = muscle fat infiltration, Post. = posterior, Ant. = anterior.

Supplementary Tables

Table S1: Details of GWAS included in Mendelian randomization analyses.

Phenotypes	Sample size	Ethnicity	Consortium	PubMed ID
**Exposures**				
Body mass index	449,889	EUR	GIANT	29273807
Waist circumference	153,932	EUR	GIANT	25673412
Waist-to-hip ratio	212,188	EUR	GIANT	25673412
Liver fat	26,992	EUR	UKB	This study
Liver volume	26,992	EUR	UKB	This study
Pancreas fat	26,208	EUR	UKB	This study
Pancreas volume	33,528	EUR	UKB	This study
Visceral adipose tissue	37,904	EUR	UKB	This study
Subcutaneous adipose tissue	37,904	EUR	UKB	This study
Visceral-to-subcutaneous adipose tissue ratio	37,904	EUR	UKB	This study
Anterior thigh fat-free muscle volume	35,106	EUR	UKB	This study
Anterior thigh muscle fat infiltration	35,106	EUR	UKB	This study
Posterior thigh fat-free muscle volume	35,152	EUR	UKB	This study
Posterior thigh muscle fat infiltration	35,084	EUR	UKB	This study
**Outcomes**				
Type 2 Diabetes	455,313	EUR	DIAGRAM	30297969
Myocardial infarction	120,575	EUR	CARDIoGRAM	28209224
Cerebral infarction	446,696	EUR	MEGASTROKE	29531354
Essential hypertension	391,981	EUR	FINNGEN	36653562
Hyperlipidemia	365,889	EUR	FINNGEN	36653562

SNPs, single nucleotide polymorphism; EUR, European; GIANT, Genetic Investigation of ANthropometric Traits; UKB, UK Biobank; DIAGRAM, (DIAbetes Genetics Replication and Meta-analysis); CARDIoGRAM (Coronary ARtery DIsease Genome-wide Replication and Meta-analysis).

Table S2. Characteristics of UK Biobank participants at initial assessment (instance 0) by sex and MRI participation status.

	Females			Males		
**Trait**	Full cohort	MRI-subset	difference	Full cohort	MRI-subset	difference
Age (years, mean ± SD)	56.7 ± 7.9	54.4 ± 7.3	-2.3***	57.1 ± 8.1	55.9 ± 7.5	-1.2***
Year of birth (year, mean ± SD)	1951.4 ± 7.9	1953.6 ± 7.3	2.2***	1951.0 ± 8.1	1952.2 ± 7.5	1.2***
Height (cm, mean ± SD)	162.6 ± 6.2	163.4 ± 6.1	0.8***	175.9 ± 6.8	176.7 ± 6.6	0.8***
Weight (kg, mean ± SD)	71.5 ± 14.0	69.6 ± 12.6	-1.9***	86.2 ± 14.3	84.9 ± 12.9	-1.3***
BMI (kg/m ≤, mean ± SD)	27.1 ± 5.2	26.1 ± 4.5	-1.0***	27.9 ± 4.2	27.2 ± 3.7	-0.7***
Waist circumference (cm, mean ± SD)	84.6 ± 12.5	81.8 ± 11.2	-2.8***	97.1 ± 11.4	94.9 ± 10.3	-2.2***
Hip circumference (cm, mean ± SD)	103.4 ± 10.3	101.8 ± 9.1	-1.6***	103.5 ± 7.6	102.8 ± 6.8	-0.7***
Waist-to-hip ratio (mean ± SD)	0.82 ± 0.07	0.80 ± 0.07	-0.02***	0.94 ± 0.07	0.92 ± 0.06	-0.02***
Smoking (Never/Previous/Current) (%)	141545/76696/21125 (59.1/32.0/8.8%)	12262/5773/963 (64.5/30.4/5.1%)	-5.4%*** (ever smokers)	98428/78924/24201 (48.8/39.2/12.0%)	10097/6389/1277 (56.8/36.0/7.2)	-7.8%*** (ever smokers)
Type 2 Diabetes (%)	4087 (1.7%)	185 (0.972%)	-0.7%***	7052 (3.49%)	376 (2.11%)	-1.4%***
Hypertension (%)	53735 (22.4%)	3145 (16.5%)	-5.9%***	62312 (30.8%)	4644 (26.1%)	-4.7%***
Myocardial infarction (%)	2018 (0.84%)	71 (0.373%)	-0.5%***	8413 (4.16%)	416 (2.34%)	-1.8%***
Hyperlipidemia (%)	27222 (11.3%)	1613 (8.47%)	-2.8%***	37842 (18.7%)	3104 (17.4%)	-1.3%***
Cerebral infarction (%)	479 (0.199%)	16 (0.0841%)	-0.1%***	966 (0.478%)	35 (0.197%)	-0.3%***

Values are presented as mean ± SD for continuous traits and number (percentage) for categorical traits. Prevalence values also refer to diagnosis before or at baseline assessment (instance 0). Differences between the MRI subset and the full cohort were assessed using t tests for continuous traits and χ^2 tests for categorical traits. Significance is denoted as $^{***}p < 0.001$.

Table S3. Self-reported ethnic background of UK Biobank participants at the initial MRI imaging session.

Ethnical background	Females (n)	Males (n)
**White British**	19,034	17,797
**Asian or Asian British**	174	296
**Black or Black British**	159	128
**Mixed**	114	74

Ethnic background was assessed via touchscreen questionnaire at the first assessment centre visit (Data Field 21000).

Table S4. Stepwise Cox proportional hazards regression to determine informative traits for the rate of incident type 2 diabetes.

**Females**				
**Variable#**	AIC#	Hazard ratio (95% CI)	P	n
**VAT**	611.3	1.87 (1.20 – 2.92)	5.99E-03	6097
**Pancreas volume**	598.1	0.64 (0.47 – 0.86)	2.84E-03	
**Liver fat %**	591.9	1.47 (0.96 – 2.26)	7.70E-02	
**Waist-to-hip ratio**	587.8	1.39 (0.97 – 1.99)	7.43E-02	
**Liver volume**	587.5	1.36 (0.91 – 2.05)	1.32E-01	
**Males**				
**Liver fat %**	1188.7	1.75 (1.34 – 2.30)	4.95E-05	5722
**Liver volume**	1173.7	1.89 (1.43 – 2.48)	5.73E-06	
**Pancreas volume**	1169.6	0.75 (0.60 – 0.93)	9.88E-03	
**VSR**	1164.9	1.34 (1.07 – 1.68)	9.86E-03	

#Variables were selected with stepwise Cox proportional hazards regression using the Akaike information criterion to determine the order in which effects enter and leave the model. Hazard ratios describe the effect of selected variables in the training subset. AIC = Akaike information criterion. VAT = Visceral adipose tissue volume. VSR = Visceral-to-subcutaneous adipose tissue mass ratio.

Table S5. Stepwise Cox proportional hazards regression to determine informative traits for the rate of incident hyperlipidemia.

**Females**				
**Variable#**	AIC#	Hazard ratio (95% CI)	P	n
**Pancreas fat %**	2152.84	1.29 (1.07 – 1.54)	5.97E-03	6097
**Liver volume**	2151.53	1.22 (1.03 – 1.43)	2.02E-02	
**VSR**	2150.59	1.16 (0.97 – 1.38)	1.02E-01	
**Males**				
**VAT**	2230.56	1.49 (1.15 – 1.93)	2.93E-03	5722
**Waist-to-hip ratio**	2217.80	1.43 (1.11 – 1.84)	5.47E-03	
**Waist circumference**	2216.52	0.77 (0.57 – 1.06)	1.11E-01	

#Variables were selected with stepwise Cox proportional hazards regression using the Akaike information criterion to determine the order in which effects enter and leave the model. Hazard ratios describe the effect of selected variables in the training subset. AIC = Akaike information criterion. VSR = Visceral-to-subcutaneous adipose tissue mass ratio. VAT = Visceral adipose tissue volume.

Table S6. Difference-in-coefficients analyses of adiposity traits on BMI-associated type 2 diabetes risk in female and male UK Biobank participants.

	Females						Males					
	N	Beta-estimate (95% CI)	P	Sobel test	Bonf.	% mediated 95% CI[#]	N	Beta-estimate (95% CI)	P1	Sobel test	Bonf.	% mediated 95% CI[#]
**BMI**	11,925	1.06 (0.85–1.27)	1.97E-22				10,851	0.78 (0.62–0.94)	8.18E-22			
**BMI**	11,925	0.38 (-0.03–0.79)	6.74E-02	6.36E-05	*	64%	10,851	0.41 (0.10–0.72)	1.06E-02	3.09E-03		48%
**WC**	11,925	0.79 (0.37–1.20)	1.95E-04			(25%–103%)	10,851	0.41 (0.09–0.72)	1.08E-02			(15%–81%)
**BMI**	11,925	0.85 (0.61–1.09)	3.41E-12	1.07E-05	*	20%	10,851	0.61 (0.41–0.80)	2.22E-09	1.95E-03		23%
**WHR**	11,925	0.66 (0.43–0.89)	1.40E-08			(5%–35%)	10,851	0.27 (0.07–0.46)	6.82E-03			(8%–38%)
**BMI**	11,925	0.24 (-0.10–0.58)	1.63E-01	6.83E-10	*	77%	10,851	0.45 (0.19–0.70)	6.36E-04	5.32E-04	*	43%
**VAT**	11,925	1.07 (0.73–1.41)	5.21E-10			(20%–134%)	10,851	0.41 (0.15–0.66)	1.67E-03			(18%–68%)
**BMI**	11,925	1.71 (1.15–2.27)	1.65E-09	9.94E-01		-61%	10,851	0.76 (0.44–1.07)	2.50E-06	4.27E-01		3%
**SAT**	11,925	-0.70 (-1.25–-0.15)	1.26E-02			(-140%–18%)	10,851	0.01 (-0.30–0.31)	9.73E-01			(-15%–21%)
**BMI**	11,925	1.09 (0.86–1.32)	1.77E-20	8.98E-01		-3%	10,851	0.81 (0.64–0.97)	8.09E-21	1.00		-3%
**VSR**	11,925	0.83 (0.60–1.06)	2.09E-12			(-15%–10%)	10,851	0.25 (0.09–0.41)	2.22E-03			(-12%–7%)
**BMI**	11,925	1.06 (0.84–1.27)	1.30E-22	2.89E-01		1%	10,851	0.77 (0.61–0.93)	1.72E-21	9.17E-02		2%
**Pancreas volume**	11,925	-0.34 (-0.56–-0.12)	2.19E-03			(-10%–11%)	10,851	-0.24 (-0.41–-0.08)	3.62E-03			(-8%–11%)
**BMI**	11,925	0.81 (0.56–1.06)	1.25E-10	9.70E-05	*	24%	10,851	0.65 (0.47–0.82)	6.02E-13	4.39E-04	*	18%
**Pancreas fat %**	11,925	0.54 (0.30–0.79)	1.74E-05			(8%–40%)	10,851	0.31 (0.14–0.49)	4.15E-04			(5%–30%)
**BMI**	11,925	0.60 (0.32–0.88)	3.26E-05	3.16E-08	*	44%	10,851	0.27 (0.07–0.46)	7.11E-03	9.11E-21	*	66%
**Liver volume**	11,925	0.70 (0.42–0.99)	1.51E-06			(18%–69%)	10,851	0.88 (0.67–1.08)	2.15E-17			(30%–102%)
**BMI**	11,925	0.65 (0.39–0.92)	1.30E-06	1.45E-07	*	38%	10,851	0.39 (0.20–0.58)	5.26E-05	8.15E-13	*	50%
**Liver fat %**	11,925	0.75 (0.49–1.01)	1.73E-08			(15%–62%)	10,851	0.72 (0.54–0.91)	1.79E-14			(22%–78%)
**BMI**	11,925	1.00 (0.74–1.27)	6.88E-14	1.98E-01		5%	10,851	0.65 (0.47–0.84)	6.44E-12	2.04E-03		17%
**Anterior thigh MFI**	11,925	0.10 (-0.16–0.36)	4.47E-01			(-10%–21%)	10,851	0.23 (0.03–0.42)	2.20E-02			(3%–30%)
**BMI**	11,925	0.98 (0.73–1.24)	6.66E-14	1.20E-01		7%	10,851	0.65 (0.46–0.84)	2.04E-11	2.59E-03		17%
**Posterior thigh MFI**	11,925	0.14 (-0.12–0.39)	2.88E-01			(-8%–22%)	10,851	0.22 (0.03–0.42)	2.24E-02			(3%–32%)

The 'difference-in-coefficients' method was used to estimate the proportion of the association between BMI and outcome explained by adiposity traits. Separate Cox proportional hazards models were constructed for all covariates selected through stepwise Cox regression. The proportion explained by the adiposity trait was considered significant when the estimated hazard ratio for BMI in the extended model was significantly reduced compared with the basic model including BMI only. BMI = body mass index. WHR = waist-to-hip ratio. VSR = visceral-to-subcutaneous adipose tissue mass ratio. HR = hazard ratio. CI = confidence interval. * Sobel tests were significant after multiple testing adjustment, indicating that a significant proportion of the BMI effect could be explained by the adiposity trait. # Confidence intervals for the proportion explained were approximated using the delta method, based on the standard errors of the indirect and total effect estimates.

Table S7. Difference-in-coefficients analyses of adiposity traits on BMI-associated hyperlipidaemia risk in female and male UK Biobank participants.

	Females						Males					
	N	Beta estimate (95% CI)	P	Sobel test	Bonf.	% mediated 95% CI#	N	Beta estimate (95% CI)	P	Sobel test	Bonf.	% mediated 95% CI#
**BMI**	10,281	0.21 (0.09–0.33)	5.03E-04				8,173	0.36 (0.25–0.47)	2.41E-11			
**BMI**	10,281	0.05 (-0.16–0.26)	6.58E-01	3.46E-02		78%	8,173	0.35 (0.14–0.56)	8.34E-04	4.57E-01		3%
**WC**	10,281	0.19 (-0.02–0.41)	7.57E-02			(-50%–205%)	8,173	0.01 (-0.19–0.21)	9.14E-01			(-10%–15%)
**BMI**	10,281	0.14 (0.01–0.27)	3.64E-02	4.89E-03		34%	8,173	0.26 (0.12–0.39)	1.83E-04	6.97E-03		29%
**WHR**	10,281	0.17 (0.04–0.29)	1.16E-02			(5%–63%	8,173	0.17 (0.04–0.30)	1.25E-02			(10%–47%)
**BMI**	10,281	-0.10 (-0.29–0.10)	3.30E-01	4.64E-05	*	145%	8,173	0.12 (-0.06–0.29)	1.95E-01	2.50E-04	*	68%
**VAT**	10,281	0.40 (0.20–0.59)	7.87E-05			(50%–240%)	8,173	0.31 (0.14–0.48)	4.68E-04			(30%–106%)
**BMI**	10,281	0.06 (-0.24–0.36)	6.93E-01	1.45E-01		71%	8,173	0.40 (0.20–0.61)	1.29E-04	6.93E-01		–12%
**SAT**	10,281	0.16 (-0.15–0.47)	3.07E-01			(-20%–160%)	8,173	-0.05 (-0.25–0.15)	6.23E-01			(-50%–26%)
**BMI**	10,281	0.18 (0.06–0.30)	4.04E-03	2.23E-03		16%	8,173	0.37 (0.26–0.48)	3.09E-11	1.00E+00		–3%
**VSR**	10,281	0.23 (0.10–0.35)	3.08E-04			(3%–28%)	8,173	0.21 (0.11–0.32)	8.04E-05			(-15%–9%)
**BMI**	10,281	0.21 (0.09–0.33)	4.90E-04	5.50E-01		0%	8,173	0.35 (0.25–0.46)	1.02E-10	2.15E-01		1%
**Pancreas volume**	10,281	-0.12 (-0.25–0.01)	6.62E-02			(-12%–12%)	8,173	0.06 (-0.05–0.18)	2.88E-01			(-8%–11%)
**BMI**	10,281	0.06 (-0.08–0.20)	3.79E-01	4.47E-05	*	71%	8,173	0.32 (0.20–0.44)	1.39E-07	7.78E-02		11%
**Pancreas fat %**	10,281	0.29 (0.15–0.43)	7.03E-05			(30%–111%)	8,173	0.09 (-0.03–0.21)	1.32E-01			(-5%–27%)
**BMI**	10,281	0.13 (-0.02–0.28)	9.48E-02	2.87E-02		40%	8,173	0.36 (0.23–0.49)	4.48E-08	5.18E-01		0%
**Liver volume**	10,281	0.14 (-0.02–0.29)	7.96E-02			(-5%–86%)	8,173	0.00 (-0.14–0.13)	9.70E-01			(-20%–19%)
**BMI**	10,281	0.15 (0.00–0.30)	4.73E-02	8.97E-02		29%	8,173	0.34 (0.21–0.47)	3.82E-07	2.91E-01		6%
**Liver fat %**	10,281	0.10 (-0.05–0.25)	2.09E-01			(0%–58%)	8,173	0.04 (-0.09–0.17)	5.73E-01			(-10%–22%)
**BMI**	10,281	0.15 (0.01–0.29)	4.20E-02	4.74E-02		31%	8,173	0.28 (0.16–0.41)	7.36E-06	6.02E-03		22%
**Anterior thigh MFI**	10,281	0.11 (-0.04–0.26)	1.39E-01			(1%–60%)	8,173	0.16 (0.03–0.29)	1.58E-02			(5%–38%)
**BMI**	10,281	0.17 (0.03–0.31)	1.54E-02	1.49E-01		18%	8,173	0.30 (0.18–0.43)	1.89E-06	4.85E-02		15%
**Posterior thigh MFI**	10,281	0.07 (-0.08–0.21)	3.79E-01			(0%–36%)	8,173	0.11 (-0.02–0.24)	1.01E-01			(3%–27%)

The 'difference-in-coefficients' method was used to estimate the proportion of the association between BMI and outcome explained by adiposity traits. Separate Cox proportional hazards models were constructed for all covariates selected through stepwise Cox regression. The proportion explained by the adiposity trait was considered significant when the estimated hazard ratio for BMI in the extended model was significantly reduced compared with the basic model including BMI only. BMI = body mass index. WHR = waist-to-hip ratio. VSR = visceral-to-subcutaneous adipose tissue mass ratio. HR = hazard ratio. CI = confidence interval. * Sobel tests were significant after multiple testing adjustment, indicating that a significant proportion of the BMI effect could be explained by the adiposity trait. # Confidence intervals for the proportion explained were approximated using the delta method, based on the standard errors of the indirect and total effect estimates.
